# Supplementary material for: Inhibitors supercharge kinase turnover through native proteolytic circuits
Source: Nature. 2025 Nov 26;649(8098):1032–41. doi: 10.1038/s41586-025-09763-9 (PMC12823440; doi:10.1038/s41586-025-09763-9)
Supplement: Supplementary file 1 — Supplementary Methods, Supplementary Tables 1–5, Supplementary Figs. 1–8 and Supplementary References. [file 41586_2025_9763_MOESM1_ESM.pdf]

nature portfolio

https://doi.org/10.1038/s41586-025-09763-9

Supplementary information

Inhibitors supercharge kinase turnover through native proteolytic circuits

In the format provided by the authors and unedited

Supplementary Methods

Synthesis of the TAK285 chemoproteomics probe

General information

All starting materials, solvents, and reagents used were obtained from commercial sources and used without further purification unless otherwise stated. Dry solvents were bought with and stored over molecular sieve. Reactions were monitored by thin-layer chromatography (TLC) using pre-coated silica gel plates F-254 and high-performance liquid chromatography - mass spectrometry (HPLC-MS) using the Agilent 1260 Infinity II series system on a Poroshell 120 EC-C18 column, the 1260 DAD HS diode array detector and InfinityLab LC/MSD mass detector running in positive electron spray ionization (ESI+) mode. Column chromatography was performed on the Interchim puriFlash XS 420Plus system using PF-30SIHP (30 µM particle size) prepacked silica gel cartridges and PF-30C18HP (30 µM particle size) prepacked reverse phase cartridges.

Proton (^{1}H) and carbon (^{13}C) nuclear magnetic resonance (NMR) spectra were recorded on the Bruker AV 400 HD (1H: 400.1 MHz, 13C: 100.8 MHz), with chemical shifts referenced to residual deuterated solvent peaks. Chemical shifts (δ) are reported in parts per million (ppm), coupling constants (J) are given in Hertz (Hz). Signal multiplicities are designated as follows: s (singlet), d (doublet), dd (doublet of doublets), t (triplet), q (quartet) and m (multiplet).

Low-resolution mass spectrometry (LR-MS) was performed using ESI on an InfinityLab LC/MSD mass detector running in ESI+ mode. High-resolution mass spectrometry (HR-MS) analyses were carried out on a Bruker Daltonik micrOTOF-QII system running in ESI+ mode.

Synthesis of ***tert*-butyl (2-bromoethyl)carbamate**:

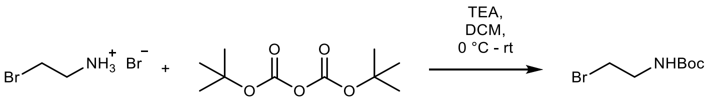

To a chilled solution of 2-bromoethylamine hydrobromide (5.00 g, 24.4 mmol, 1.1 equiv.) in dry DCM (20 mL) was added di-*tert*-butyl dicarbonate (4.84 g, 22.2 mmol, 1.0 equiv.) followed by TEA (4.5 mL, 33 mmol, 1.5 equiv.), the mixture was allowed to warm to rt overnight. The reaction mixture was washed with water (20 mL) and all volatiles were removed *in vacuo* to produce the title compound as a colorless oil (4.98 g, quant.). The product was used without further purification.

^{1}H-NMR (400 MHz, DMSO-d_6): δ = 7.08 (t, $^3J_{HH}$ = 6.0 Hz, 1H), 3.42 (t, $^3J_{HH}$ = 6.7 Hz, 2H), 3.32 – 3.26 (m, 2H), 1.38 (s, 9H) ppm.

Synthesis of ***tert*-butyl (2-(4-chloro-5*H*-pyrrolo[3,2-*d*]pyrimidin-5-yl)ethyl)carbamate**:

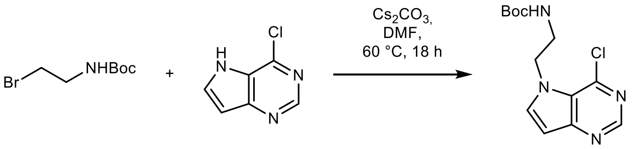

To a solution of 4-chloro-5*H*-pyrrolo[3,2-*d*]pyrimidine (571 mg, 3.72 mmol, 1.0 equiv.) in dry DMF (50 mL) was added Cs_2CO_3 (1.82 g, 5.58 mmol, 1.5 equiv.) followed by *tert*-butyl (2-bromoethyl)carbamate (1.00 g, 4.46 mmol, 1.2 equiv.), the mixture was heated to 60 °C for 3 days. Half of the solvent was removed *in vacuo*, ethyl acetate (50 mL) was added and the solution was washed thrice with water (20 mL each). The organic phase was dried over $MgSO_4$ and all

volatiles were removed *in vacuo*. The crude was purified by reverse phase flash column chromatography (H_2O/ACN 98/2 – 0/100) to produce the title compound as an off-white solid (708 mg, 2.39 mmol, 64%).

^{1}H-NMR (400 MHz, DMSO-*d*$_6$): δ = 8.60 (s, 1H), 7.86 (d, $^3J_{HH}$ = 3.2 Hz, 1H), 6.90 (s, 1H), 6.72 (d, $^3J_{HH}$ = 3.2 Hz, 1H), 4.51 (t, $^3J_{HH}$ = 5.7 Hz, 2H), 3.36 (q, $^3J_{HH}$ = 6.0 Hz, 2H), 1.26 (s, 9H) ppm.
ESI-MS (+): *m/z* calculated for $C_{13}H_{17}ClN_4O_2$ $[M+H]^+$: 297.1; found *m/z* = 297.1 $[M+H]^+$

Synthesis of **2-chloro-4-nitro-1-(3-(trifluoromethyl)phenoxy)benzene**:

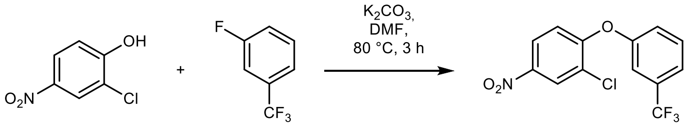

To a solution of 2-chloro-4-nitrophenol (2.00 g, 11.4 mmol, 1.0 equiv.) in dry DMF (40 mL) was added K_2CO_3 (2.36 g, 17.1 mmol, 1.5 equiv.) followed by 1-fluoro-3-(trifluoromethyl)benzene (1.85 g, 11.4 mmol, 1.0 equiv.) and the mixture was heated to 80 °C for 3 h. The product was precipitated by adding water (40 mL), filtered off, washed with water (40 mL) and dried *in vacuo* to produce the title compound (3.01 g, 10.5 mmol, 92%) as a colorless solid. The product was used without further purification.

^{1}H-NMR (400 MHz, DMSO-*d*$_6$): δ = 8.50 (d, $^4J_{HH}$ = 2.8 Hz, 1H), 8.21 (dd, $^3J_{HH}$ = 9.2, $^4J_{HH}$ = 2.7 Hz, 1H), 7.77 – 7.69 (m, 1H), 7.66 (d, $^3J_{HH}$ = 7.8 Hz, 1H), 7.60 (s, 1H), 7.52 – 7.46 (m, 1H), 7.21 (d, $^3J_{HH}$ = 9.1 Hz, 1H) ppm.
ESI-MS (+): *m/z* calculated for $C_{13}H_7ClF_3NO_3$ $[M+H]^+$: 318.0; found *m/z* = 318.0 $[M+H]^+$

Synthesis of **3-chloro-4-(3-(trifluoromethyl)phenoxy)aniline**:

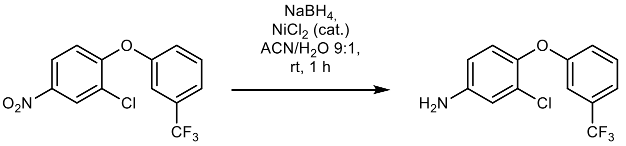

To a solution of 2-chloro-4-nitro-1-(3-(trifluoromethyl)phenoxy)benzene (2.00 g, 6.30 mmol. 1.0 equiv.) in ACN/H_2O (9:1, 50 mL) was added $NiCl_2$ (163 mg, 1.26 mmol, 0.2 equiv.) followed by $NaBH_4$ (716 mg, 18.9 mmol, 3.0 equiv.) in portions. After the gas formation stopped the mixture was stirred for one hour at rt before filtering the mixture through a silica plug and flushing the plug with EtOH (100 mL). All volatiles were removed *in vacuo* and the crude was purified by flash column chromatography (hexanes/ethyl acetate 100/0 – 0/100) to produce the title compound as an off white solid (1.70 g, 5.92 mmol, 94%).

^{1}H-NMR (400 MHz, DMSO-*d*$_6$): δ = 7.55 (t, $^3J_{HH}$ = 8.0 Hz, 1H), 7.37 (d, $^3J_{HH}$ = 7.7 Hz, 1H), 7.12 – 7.04 (m, 2H), 6.99 (d, $^3J_{HH}$ = 8.7 Hz, 1H), 6.75 (d, $^4J_{HH}$ = 2.6 Hz, 1H), 6.59 (dd, $^3J_{HH}$ = 8.7, $^4J_{HH}$ = 2.6 Hz, 1H), 5.41 (s, 2H) ppm.
ESI-MS (+): *m/z* calculated for $C_{13}H_9ClF_3NO$ $[M+H]^+$: 288.0; found *m/z* = 288.1 $[M+H]^+$

Synthesis of **5-(2-aminoethyl)-*N*-(3-chloro-4-(3-(trifluoromethyl)phenoxy)phenyl)-5*H*-pyrrolo[3,2-*d*]-pyrimidin-4-amine**:

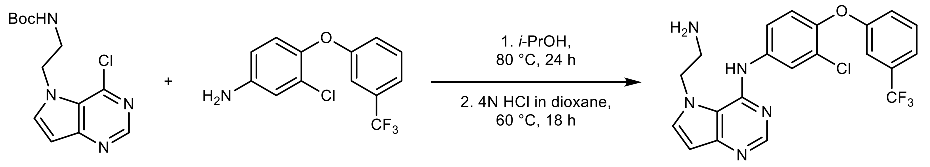

To a solution of *tert*-butyl (2-(4-chloro-5*H*-pyrrolo[3,2-*d*]pyrimidin-5-yl)ethyl)carbamate (500 mg, 1.68 mmol, 1.0 equiv.) in dry *i*-PrOH (10 mL) was added 3-chloro-4-(3-

(trifluoromethyl)phenoxy)aniline (535 mg, 1.85 mmol, 1.1 equiv.) and the mixture was heated to 80 °C for 24 h. All volatiles were removed *in vacuo*. The intermediate was dissolved in 4N HCl in dioxane (10 mL) and heated to 60 °C for 18 h. The resulting precipitate was filtered off, washed with dioxane (30 mL) and dried *in vacuo*. The crude was purified by reverse phase flash column chromatography (H_2O/ACN 98/2 – 0/100) to produce the title compound as an off white solid (541 mg, 1.20 mmol, 72% over 2 steps).

^{1}H-NMR (400 MHz, DMSO-d_6): δ = 8.32 (s, 1H), 8.06 (d, $^4J_{HH}$ = 2.6 Hz, 1H), 7.73 (dd, $^3J_{HH}$ = 8.8, $^4J_{HH}$ = 2.6 Hz, 1H), 7.65 – 7.58 (m, 2H), 7.45 (d, $^3J_{HH}$ = 7.9 Hz, 1H), 7.29 (d, $^3J_{HH}$ = 8.8 Hz, 1H), 7.20 (dd, $^3J_{HH}$ = 4.9, $^4J_{HH}$ = 2.6 Hz, 2H), 6.49 (d, $^4J_{HH}$ = 3.1 Hz, 1H), 6.01 (s, 2H), 4.42 – 4.32 (m, 2H), 3.11 – 3.03 (m, 2H).ppm.
ESI-MS (+): *m/z* calculated for $C_{21}H_{17}ClF_3N_5O$ $[M+H]^+$: 448.1; found *m/z* = 448.1 $[M+H]^+$

Synthesis of **benzyl (6-((2-(4-((3-chloro-4-(3-(trifluoromethyl)phenoxy)phenyl)amino)-5*H*-pyrrolo-[3,2-*d*]pyrimidin-5-yl)ethyl)amino)hexyl)carbamate**:

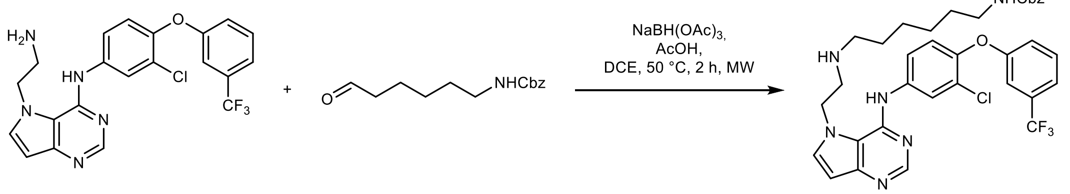

To a solution of 5-(2-aminoethyl)-*N*-(3-chloro-4-(3-(trifluoromethyl)phenoxy)phenyl)-5*H*-pyrrolo[3,2-*d*]-pyrimidin-4-amine (200 mg, 447 µmol, 1.0 equiv.) in dry DCE (3.0 mL) was added AcOH (51.1 µL, 893 µmol, 2.0 equiv.), benzyl (6-oxohexyl)carbamate (111 mg, 447 µmol, 1.0 equiv.) and lastly $NaBH(OAc)_3$ (190 mg, 893 µmol, 2.0 equiv.). The mixture was heated to 50 °C for 2 h by microwave irradiation. Upon completion (monitored by HPLC) all volatiles were removed *in vacuo* and the crude was purified by reverse phase flash column chromatography (H_2O/ACN 98/2 – 0/100) to produce the title compound (180 mg, 264 µmol, 59%).

^{1}H-NMR (400 MHz, DMSO-d_6): δ = 8.33 (s, 1H), 8.02 (d, $^4J_{HH}$ = 2.6 Hz, 1H), 7.70 (dd, $^3J_{HH}$ = 8.8, $^4J_{HH}$ = 2.6 Hz, 1H), 7.65 (d, $^4J_{HH}$ = 3.1 Hz, 1H), 7.60 (t, $^3J_{HH}$ = 8.0 Hz, 1H), 7.48 – 7.42 (m, 1H), 7.37 – 7.25 (m, 6H), 7.24 – 7.16 (m, 2H), 7.13 (t, $^3J_{HH}$ = 5.7 Hz, 1H), 6.50 (d, $^4J_{HH}$ = 3.1 Hz, 1H), 4.97 (s, 2H), 4.49 – 4.40 (m, 2H), 3.05 – 2.97 (m, 2H), 2.88 (q, $^3J_{HH}$ = 6.6 Hz, 2H), 2.35 (t, $^3J_{HH}$ = 7.3 Hz, 2H), 1.33 – 1.19 (m, 4H), 1.17 – 1.00 (m, 4H).ppm.
ESI-MS (+): *m/z* calculated for $C_{35}H_{36}ClF_3N_6O_3$ $[M+H]^+$: 681.2; found *m/z* = 681.2 $[M+H]^+$

Synthesis of **benzyl (6-(*N*-(2-(4-((3-chloro-4-(3-(trifluoromethyl)phenoxy)phenyl)amino)-5*H*-pyrrolo-[3,2-*d*]pyrimidin-5-yl)ethyl)-3-hydroxy-3-methylbutanamido)hexyl)carbamate**:

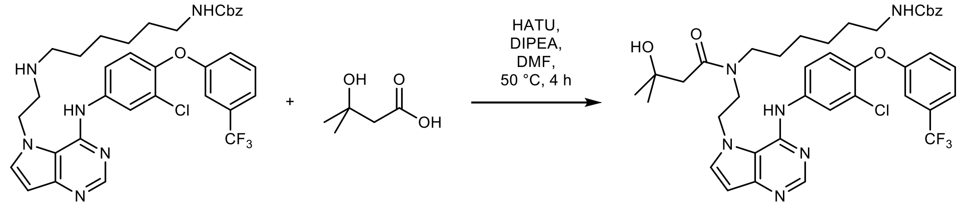

To a solution of benzyl (6-((2-(4-((3-chloro-4-(3-(trifluoromethyl)phenoxy)phenyl)amino)-5*H*-pyrrolo-[3,2-*d*]pyrimidin-5-yl)ethyl)amino)hexyl)carbamate (65 mg, 95 µmol, 1.0 equiv.) in dry DMF (2 mL) was added 3-hydroxy-3-methylbutanoic acid (12.4 mg, 105 µmol, 1.1 equiv.), HATU (44 mg, 110 µmol, 1.20 equiv.) and DIPEA (25 µL, 140 µmol, 1.5 equiv.), the solution was heated to 50 °C for 4 h. All volatiles were removed *in vacuo*. The crude was dissolved in ethyl acetate (10 mL) and washed twice with water (5 mL each), the organic phase was dried over $MgSO_4$ and all volatiles were removed *in vacuo* to produce the title compound (48 mg, 61 µmol, 64%) as a brown oil. The product was used without further purification.

¹H-NMR (400 MHz, DMSO-*d*₆): δ = 9.14 (s, 1H), 8.34 (s, 1H), 8.09 (d, $^4J_{HH}$ = 2.6 Hz, 1H), 7.91 (dd, $^3J_{HH}$ = 8.9, $^4J_{HH}$ = 2.6 Hz, 1H), 7.75 – 7.70 (m, 2H), 7.65 – 7.56 (m, 2H), 7.50 – 7.43 (m, 2H), 7.39 – 7.26 (m, 5H), 7.26 – 7.17 (m, 3H), 6.51 (d, *J* = 3.1 Hz, 1H), 4.70 (d, *J* = 6.8 Hz, 1H), 4.53 (t, $^3J_{HH}$ = 7.9 Hz, 2H), 3.65 – 3.52 (m, 2H), 3.28 (t, $^3J_{HH}$ = 7.7 Hz, 2H), 3.02 – 2.89 (m, 3H), 2.46 (s, 2H), 1.52 – 1.28 (m, 6H), 1.20 (s, 6H), 0.88 (s, 2H) ppm.
ESI-MS (+): *m/z* calculated for $C_{40}H_{44}ClF_3N_6O_5$ $[M+H]^+$: 781.3; found *m/z* = 781.3 $[M+H]^+$

Synthesis of ***N*-(6-aminohexyl)-*N*-(2-(4-((3-chloro-4-(3-(trifluoromethyl)phenoxy)phenyl)amino)-5*H*-pyrrolo[3,2-*d*]pyrimidin-5-yl)ethyl)-3-hydroxy-3-methylbutanamide (**amine-tethered TAK285 probe**):**

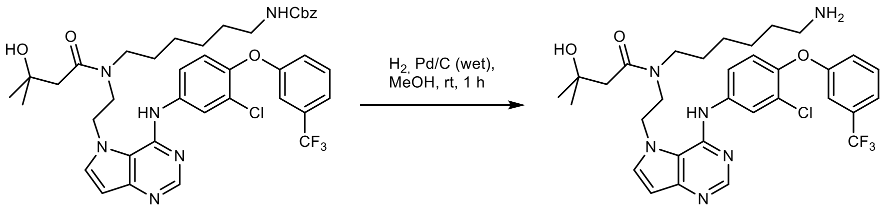

A solution of benzyl (6-(*N*-(2-(4-((3-chloro-4-(3-(trifluoromethyl)phenoxy)phenyl)amino)-5*H*-pyrrolo-[3,2-*d*]pyrimidin-5-yl)ethyl)-3-hydroxy-3-methylbutanamido)hexyl)carbamate (24 mg, 31 µmol, 1.0 equiv.) in dry MeOH (5 mL) was thoroughly degassed using an argon filled balloon and sonication before adding Pd/C (wet) (5 mg, 20wt%) and changing the atmosphere to H_2. The mixture was stirred at rt for 1 h after that the mixture was filtered through a silica plug and the plug flushed with MeOH (20 mL). All volatiles were removed *in vacuo* and the crude was purified by reverse phase flash column chromatography (H_2O/ACN 98/2 – 0/100) to produce the title compound as a brown oil (7 mg, 10 µmol, 35%).

¹H-NMR (400 MHz, DMSO-*d*₆): δ = 9.11 (s, 1H), 8.33 (s, 1H), 8.08 (s, 1H), 7.93 – 7.86 (m, 1H), 7.74 – 7.67 (m, 1H), 7.62 (t, $^3J_{HH}$ = 8.8 Hz, 1H), 7.47 (d, $^3J_{HH}$ = 8.8 Hz, 1H), 7.33 – 7.24 (m, 1H), 7.24 – 7.18 (m, 2H), 6.56 – 6.48 (m, 1H), 7.74 – 7.67 (m, 1H), 4.70 (s, 1H), 4.55 (t, $^3J_{HH}$ = 8.7 Hz, 2H), 3.63 – 3.55 (m, 2H), 3.32 – 3.23 (m, 2H), 3.10 – 3.04 (m, 1H), 2.60 (q, $^3J_{HH}$ = 7.1 Hz, 1H), 2.56 – 2.52 (m, 1H), 1.90 – 1.79 (m, 2H), 1.50 – 1.22 (m, 6H), 1.20 (s, 6H), 1.05 – 0.99 (m, 2H) ppm.

¹³C-NMR (100 MHz, DMSO-*d*₆): δ = 172.4, 157.9, 157.9, 144.5, 131.4, 130.8, 130.5, 125.0, 124.8, 123.2, 122.6, 122.5, 122.1, 120.0, 119.3, 114.5, 112.4, 101.4, 69.0, 68.6, 48.7, 48.2, 48.1, 45.2, 44.2, 44.1, 43.0, 29.6, 29.3, 26.3, 25.9, 13.9 ppm.

ESI-MS (+): *m/z* calculated for $C_{32}H_{38}ClF_3N_6O_3$ $[M+H]^+$: 647.3; found *m/z* = 647.3 $[M+H]^+$

HR-ESI-MS (+): *m/z* calculated for $C_{32}H_{38}ClF_3N_6O_3$ $[M+H]^+$: 647.2719; found *m/z* = 647.2737 $[M+H]^+$

Corresponding spectra can be found in **Supplementary Fig S3**.

Supplementary Tables S1-S5

Table S1 Primers for mutagenesis

Vector name	Primer fw	Primer rev	Method
pENTR223 EGFR T790M	TGCAACTCATCATGCAGCTCATGCCCTTC	GAAGGGCATGAGCTGCATGATGAGTTGCA	PCR
pENTR223 EGFR G719S	AAGATCAAAGTGCTGAGCTCCGGTGCGTTCG	CGAACGCACCGGAGCTCAGCACTTTGATCTT	PCR
pENTR223 EGFR L858R	CACAGATTTTGGGCGGGCCAAACTGCTGGGTGCGGAAG	CAGCAGTTTGGCCCGCCCAAAATCTGTGATCTTGACATGCT GCG	PCR
pENTR223 EGFR L861Q	GCTGGCCAAACAGCTGGGTGCGGAAGAGAAAGAATAC	TCCGCACCCAGCTGTTTGGCCAGCCCAAAATCTGTG	PCR
pENTR223 EGFR L718V	AAGATCAAAGTGGTGGGCTCCGGTGCGTTCG	CGAACGCACCGGAGCCCACCACTTTGATCTT	PCR
pENTR223 BRAF V600E	GATTTTGGTCTAGCTACAGAGAAATCTCGATGGAGTG	CACTCCATCGAGATTTCTCTGTAGCTAGACCAAAATC	PCR
pENTR223 RAF1 S256L	CTCTCCCAGAGGCAGAGGCTGACATCCACACCTAATGTCCA C	CATTAGGTGTGGATGTCAGCCTCTGCCTCTGGGAGAGGGAA C	PCR
pENTR223 RET M918T	GATTCCAGTTAAATGGACGGCAATTGAATCCCTTTTTGATCAT ATC	TCAAAAAGGGATTCAATTGCCGTCCATTTAACTGGAATCCGA CCCTG	PCR
pENTR223 ERBB4 R711C	CAGCACCCAATCAAGCTCAACTTTGTATTTTGAAAGAAAC	GTTTCTTTCAAAATACAAAGTTGAGCTTGATTGGGTGCTG	PCR
pENTR233 LYN Y32A	GCCGTGAGAGATCCAACGTCCAATAAAC	AATAGTTCTTTCAGTATTACGTACTG	SDM
pENTR233 LYN Y316A	GGAGCCCATTGCGATCATCACCGAGTAC	TCCCTGGTGACCACAG	SDM
pENTR233 LYN T319I	TCGAGTACATGGCCAAGGGCAG	TGATGATGTAAATGGGCTCCTCCCTG	SDM
pENTR233 CSK T266I	TGGAGTACATGGCCAAGGGGGAG	TGACGATGTAGAGCCCGCCCTTC	SDM
pENTR223 BLK S24A	GGGCCAATGGGCGCCCCTGAAGG	TTGTCCTTCTCTTTGATCG	SDM
pENTR223 BLK T48A	CTTCAACCACCTTGCTCCTCCACCGCCCGATGAAC	CATCGGGCGGTGGAGGAGCAAGGTGGTTGAAGACAACCAG G	PCR
pENTR223 BLK S29A	CCTGAAGGTCGCGGCCCAAGACAAGGAC	GGGCTCCATTGGCCC	PCR
pENTR223 BLK C319S	AGCCTGCTGGATTTCCTGAAGACAGATG	TCCTCTGGCCATGTACTCGGTGAC	SDM
pENTR223 RIPK2 ΔCARD	GTGGTTTCTAGATCACCATC	GGCTATACCAGGCTGCAGAC	SDM
pENTR223 RIPK2 sgRNA resistant	CAAGACATGCCGACTGGAGAGTCCAGGTGGCCGTGAAGCA C	GACTCTCCAGTCGGCATGTCTTGCGGACGACACAGTGCCA G	SDM
pENTR223 RIPK2 I212D sgRNA resistant	GACTATAGCTATGCAGTTATCACATGGGAAG	ATCGTGCTTGATACTGGCCCTTG	SDM
pENTR223 HER2 C805S	GCCCTATGGCAGCCTCTTAGACC	ATAAGCTGTGTCACCAGCTG	SDM

SDM = site directed mutagenesis, PCR = direct PCR to transformation see section **Plasmids and cloning**

Table S2 Gibson primer sequences

Vector name	Primer fw	Primer rev	Vector BB
pRRL_SFFV_LYN*_BFP_P 2A_mCherry	CGCCAGTCCTCCGAGTCGACGGCATGGGATGTATAAAATCA AAAGGGAA	CCCGAGCCACCTCCGGATCCAGGCTGCTGCTGGTATTGC	SR
pRRL_SFFV_CSK*_EF1a_i RFP670	CGGCGCGCCAGTCCTCCGAGTCGACCATGTCAGCAATACA GGCCG	CTGACGGGCACCGGAGCCACTCGAGTCACAGGTGCAGCT CGTG	iRFP670
pRRL_SFFV_BLK*_BFP_P 2A_mCherry	CGCCAGTCCTCCGAGTCGACGGCACCATGGGGCT	CCCGAGCCACCTCCGGATCCGGGCTGCAGCTCGTACTG	SR
pRRL_SFFV_ABL_BFP_P2 A_mCherry	CGCCAGTCCTCCGAGTCGACACCATGTTGGAGATCTGCCTG	CCCGAGCCACCTCCGGATCCCCTCTGCACTATGTCACTGAT TTCCT	SR
pRRL_SFFV_ABL_C464W _BFP_P2A_mCherry	CGCCAGTCCTCCGAGTCGACACCATGTTGGAGATCTGCCTG<br>GCGCCCAGAAGGCTGGCCAGAGAAGGTCTATGAACTCATG C	CCTTCTCTGGCCAGCCTTCTGGGCGCTCCAT<br>CCCGAGCCACCTCCGGATCCCCTCTGCACTATGTCACTGAT TTCCT	SR
pRRL_SFFV_BLK_S5A_BF P_P2A_mCherry	CGCCAGTCCTCCGAGTCGACGGCACCATGGGGCTGGTAG CGAGCAAAAAGCCGGACAAGGAAAAG	CCCGAGCCACCTCCGGATCCGGGCTGCAGCTCGTACTG	SR
pRRL_SFFV_BLK_S6A_BF P_P2A_mCherry	CGCCAGTCCTCCGAGTCGACGGCACCATGGGGCTGGTAAG TGCGAAAAAGCCGGACAAGGAAAAG	CCCGAGCCACCTCCGGATCCGGGCTGCAGCTCGTACTG	SR
pRRL_SFFV_SRC_BFP_P2 A_mCherry	CGGCGCGCCAGTCCTCCGAGTCGACGCCACCATGGGTAG CAACAAGAGCAAGC	CCCGAGCCACCTCCGGATCCGAGGTTCTCCCCGGGCTG	SR
pRRL_SFFV_BLK_1-20_BFP_P2A_mCherry	CGCCAGTCCTCCGAGTCGACGGCACCATGGGGCT	CCCGAGCCACCTCCGGATCCCTTGTCCTTCTCTTTGATCGG C	SR
pRRL_SFFV_BLK_1-40_BFP_P2A_mCherry	CGCCAGTCCTCCGAGTCGACGGCACCATGGGGCT	CCCGAGCCACCTCCGGATCCGGGCGGCAGTGGCGGGG	SR
pRRL_SFFV_BLK_1-57_BFP_P2A_mCherry	CGCCAGTCCTCCGAGTCGACGGCACCATGGGGCT	CCCGAGCCACCTCCGGATCCATCCAGGTGTTCATCGGGCG	SR
pRRL_SFFV_BLK_G2I_BF P_P2A_mCherry	CGCCAGTCCTCCGAGTCGACGGCACCATGATCCTGGTAAG TAGCAAAAAGCCGGACAAGG	CCCGAGCCACCTCCGGATCCGGGCTGCAGCTCGTACTG	SR
pRRL_SFFV_BLK_G2L_BF P_P2A_mCherry	CGCCAGTCCTCCGAGTCGACGGCACCATGCTGCTGGTAAG TAGCAAAAAGCCGGACAAGG	CCCGAGCCACCTCCGGATCCGGGCTGCAGCTCGTACTG	SR

pRRL_SFFV_BLK_L3A_BFP_P2A_mCherry	CGCCAGTCCTCCGAGTCGACGGCACCATGGGGGCCGTAAGTAGCAAAAAGCCGGACAAGG	CCCGAGCCACCTCCGGATCCGGGCTGCAGCTCGTACTG	SR
pRRL_SFFV_BLK_L3G_BFP_P2A_mCherry	CGCCAGTCCTCCGAGTCGACGGCACCATGGGGGGCGTAAGTAGCAAAAAGCCGGACAAGG	CCCGAGCCACCTCCGGATCCGGGCTGCAGCTCGTACTG	SR
pRRL_SFFV_BLK_L3V_BFP_P2A_mCherry	CGCCAGTCCTCCGAGTCGACGGCACCATGGGGGTGGTAAGTAGCAAAAAGCCGGACAAGG	CCCGAGCCACCTCCGGATCCGGGCTGCAGCTCGTACTG	SR
pRRL_SFFV_BLK_V4S_BFP_P2A_mCherry	CGCCAGTCCTCCGAGTCGACGGCACCATGGGGCTGTCGAGTAGCAAAAAGCCGGACAAGGAAAAG	CCCGAGCCACCTCCGGATCCGGGCTGCAGCTCGTACTG	SR
pRRL_SFFV_BLK_G2I_3xFLAG_EF1as_BFP	CGCCAGTCCTCCGAGTCGACGGCACCATGATCCTGGTAAGTAGCAAAAAGCCGGACAAGG	CCCGAGCCACCTCCGGATCCGGGCTGCAGCTCGTACTG	FLAG
pRRL_SFFV_BLK_G2L_3xFLAG _EF1as_BFP	CGCCAGTCCTCCGAGTCGACGGCACCATGCTGCTGGTAAGTAGCAAAAAGCCGGACAAGG	CCCGAGCCACCTCCGGATCCGGGCTGCAGCTCGTACTG	FLAG
pRRL_SFFV_BLK_L3A_3xFLAG _EF1as_BFP	CGCCAGTCCTCCGAGTCGACGGCACCATGGGGGCCGTAAGTAGCAAAAAGCCGGACAAGG	CCCGAGCCACCTCCGGATCCGGGCTGCAGCTCGTACTG	FLAG
pRRL_SFFV_BLK_L3G_3xFLAG _ EF1as_BFP	CGCCAGTCCTCCGAGTCGACGGCACCATGGGGGGCGTAAGTAGCAAAAAGCCGGACAAGG	CCCGAGCCACCTCCGGATCCGGGCTGCAGCTCGTACTG	FLAG
pRRL_SFFV_BLK_L3V_3xFLAG _ EF1as_BFP	CGCCAGTCCTCCGAGTCGACGGCACCATGGGGGTGGTAAGTAGCAAAAAGCCGGACAAGG	CCCGAGCCACCTCCGGATCCGGGCTGCAGCTCGTACTG	FLAG
pRRL_SFFV_BLK_V4S_3xFLAG _EF1as_BFP	CGCCAGTCCTCCGAGTCGACGGCACCATGGGGCTGTCGAGTAGCAAAAAGCCGGACAAGGAAAAG	CCCGAGCCACCTCCGGATCCGGGCTGCAGCTCGTACTG	FLAG
pRRL_SFFV_SRC_BLK_FUS1_BFP_P2A_mCherry	CGGCGCGCCAGTCCTCCGAGTCGACGCCACCATGGGTAGCAACAAGAGCAAGC	ACCACGAAATGCTTGTCTTCGGCCAGCGGGCCCGCC	SR
	GAAGACAAGCATTTCGTGGTGGC	CCCGAGCCACCTCCGGATCCGGGCTGCAGCTCGTACTG	
pRRL_SFFV_BLK_SRC_FUS2_BFP_P2A_mCherry	CGCCAGTCCTCCGAGTCGACGGCACCATGGGGCTGGTAAGTAG	CACAAAGGTGGTCACTCCACCATCCAGGTGTTCATCGGGCG	SR
	CGCCCGATGAACACCTGGATGGTGGAGTGACCACCTTTGTG	CCCGAGCCACCTCCGGATCCGAGGTTCTCCCCGGGCTG	
pRRL_SFFV_BLK_L3S,V4N_BFP_P2A_mCherry	CGCCAGTCCTCCGAGTCGACGGCACCATGGGGAGCAACAAGAGCAAAAAGCCGGACAAGGAAAAG	CCCGAGCCACCTCCGGATCCGGGCTGCAGCTCGTACTG	SR
pRRL_SFFV_SRC_S3L,N4V_BFP_P2A_mCherry	CGGCGCGCCAGTCCTCCGAGTCGACGCCACCATGGGTCTGGTAAGTAGCAAGCCCAAGGATGCCAGC	CCCGAGCCACCTCCGGATCCGAGGTTCTCCCCGGGCTG	SR
pRRL_SFFV_RIPK2*_BFP_P2A_mCherry	CGCCAGTCCTCCGAGTCGACGGCACCATGAACGGGG	CCCGAGCCACCTCCGGATCCGGGCTGCAGCTCGTACTG	SR

BB = backbone ; SR = stability reporter (pRRL_SFFV_empty_BFP_P2A_mCherry); iRFP670 = pRRL_SFFV_DCAF16_EF1a_iRFP670; * or mutated versions thereof

Table S3 Annealed oligos

Vector name	Primer fw	Primer rev	Vector BB
pRRL_SFFV_BLK_1-7_BFP_P2A_mCherry	TCGACATGGGGCTGGTAAGTAGCAAAG	GATCCTTTGCTACTTACCAGCCCCATG	SR

Table S4 sgRNA sequences

KO	sgRNA sequence 1	sgRNA sequence 2 (dual only)	Dox
CBL	GCAGGTCTAAGATATAAGGT	–	96h
CBL-B	GTCCCAAACACATCCCGA	–	96h
AAVS1	GCTGTGCCCCGATGCACAC	–	96h
CBL	GCCAGGATGAGTTACAGCA	GCTGTGCCCCGATGCACAC	1 week
CBL-B	GCTCCGGAAAGAGCATCCT	GTTGCACTCGATTGGGACAG	1 week
CBL & CBL-B	GCCAGGATGAGTTACAGCA	GTTGCACTCGATTGGGACAG	1 week
AAVS1	GCTCCGGAAAGAGCATCCT	GCTGTGCCCCGATGCACAC	1 week
PSENEN	GTTGACCAACCAGAGAAA	–	1 week
TMUB1 (1)	GGAGCCAATGGTGTCGTG	–	96h
TMUB1 (2)	GCACGAGGGGCTCCTGCG	–	96h
PSMB5 (1)	TTTGTACTGATACACCATGT	–	48h
PSMB5 (2)	GCTTCATGGAACAACCACCC	–	48h
FIP200 (1)	TTTCTAACAGCTCTATTACG	–	96h
FIP200 (2)	ACTACGATTGACACTAAAGA	–	96h
MAP2K5 (1)	GGAACAAATGCTTATATGG	–	120h
MAP2K5 (2)	GATATGTACAGAATTCATGGA	–	120h
XIAP	GCTGTGCCCCGATGCACAC	TGCGGTGCTTTAGTTGTCAT	96h
cIAP1	GTTAATGGCCACATATGGT	GCTGTGCCCCGATGCACAC	96h
XIAP and cIAP1	GTTAATGGCCACATATGGT	TGCGGTGCTTTAGTTGTCAT	96h

Table S5: Reagents and resources for MS experiments of sections "Sample preparation for full proteome profiling", "LC-MS/MS data acquisition of BioID and full proteome samples", "Data analysis and representation of BioID data" and "Processing and data analysis of full proteome profiling data"

REAGENT or RESOURCE	SOURCE	IDENTIFIER
LiChrosolv® Water	MERCK KgaA, Germany	# 1.15333.2500
PMSF	BioChemica	# A0999.0005
Protease inhibitor cocktail	Sigma-Aldrich, Germany	# P8340
SDS	SERVA Electrophoresis GmbH, Germany	#20765
HEPES	Merck, KgaA, Germany	# 1.10110.0250
Covaris S2 high performance ultrasonicator	Covaris, USA	NA
Dithiothreitol, for molecular biology, minimum 99% titration	Sigma-Aldrich, Germany	# D9779-5G
Urea	Sigma-Aldrich, Germany	# U0631-500G
Tris(hydroxymethyl)aminomethane (Tris/HCl) ultrapure grade ≥99.9%	Sigma-Aldrich, Germany	# 154563
Microcon 30, Ultracel YM-30	Merck Millipore, Ireland	# MRCF0R030
Iodoacetamide	Sigma-Aldrich, Germany	# I1149-5G
Triethylammonium Bicarbonate Buffer (TEAB), 1 M, pH 8.5	Sigma-Aldrich, Germany	# 17902
Sequencing Grade Modified Trypsin	Madison, WI, USA	# V5113
NaCl	Sigma-Aldrich, Germany	# S7653
PEPTIDE DESALTING COLUMNS 50PC	Thermo Fisher Scientific, USA	#89851
TMTpro 18-plex label reagent set	Thermo Fisher Scientific, USA	# A52045
Agilent 1200 series	Agilent	NA
150 x 2.0 mm Gemini-NX, 3 µm C18 110Å	Phenomenex, Torrance, USA	NA
TFA Uvasol (trifluoroacetic acid)	MERCK KgaA, Germany	# 1.08262.0100
0.1% TFA	Sigma-Aldrich, Germany	# 34978-2.5L-R
Orbitrap Fusion Lumos Tribrid mass spectrometer	Thermo Fisher Scientific, USA	NA
**MS data acquisition**		
Dionex Ultimate 3000 RSLCnano system	Thermo Fisher Scientific, USA	NA
Nanospray Flex Ion Source	Thermo Fisher Scientific, USA	NA
PepMap 100 C18, 5 µm, 5 × 0.3 mm	Thermo Fisher Scientific, USA	NA
Fused Silica Capillary ID 75 µm, 50 cm length	Polymicro Technologies LLC	TSP075150
ESI Emitter Fused Silica: 20 µm ID x 7 cm L x 365 µm OD; Orifice ID: 10 µm	CoAnn Technologies, USA	TIP1002010C-5
ReproSil-Pur 120 C18-AQ, 3 µm	Dr. Maisch, Germany	# r13.aq.
Suprapur ® Formic acid 98-100%	Merck KgaA, Germany	# 1.11670.1000
Acetonitril for HPLC LC-MS Grade	VWR Chemical, USA	# 83640.32
**Data processing and analysis**		
Discoverer version: v.2.4.1.15	Thermo Fisher Scientific	NA
SAINTq	Teo G, et al.[1]	NA
R version 4.3.1	The R Foundation	NA

Supplementary Fig S1. Original immunoblots

The panels have been annotated in the following way: Uncropped images are shown as overlay of luminescent and colorimetric images. Replicates 2 and 3 have been annotated for clarity. Ladders are labelled with numeric values corresponding to the size (in kDA). For originals, the blots are labelled with their corresponding antibody. Labelled blots include additional information for each depicted band e.g. for protein fusions. Saturated pixels are depicted in red.

Uncropped immunoblots and further replicates to Fig 2b.

Replicate 1 (original)

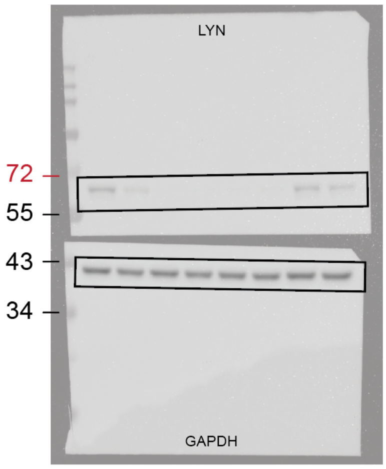

Replicate 2 (original)

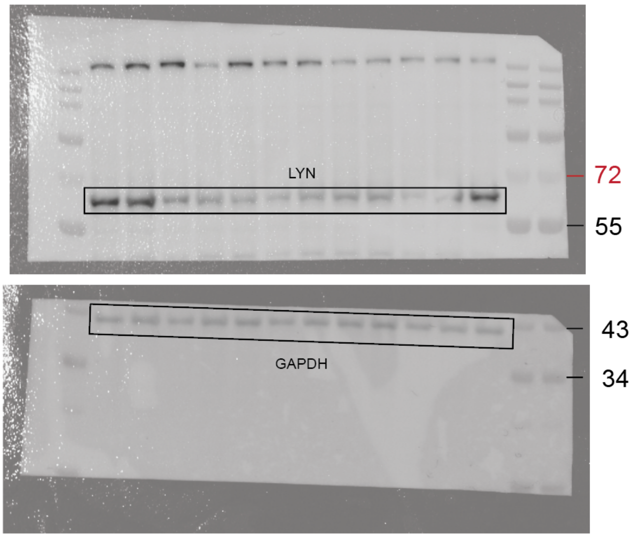

Replicate 2 (annotated blot)

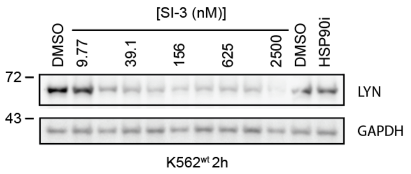

Replicate 3 (original)

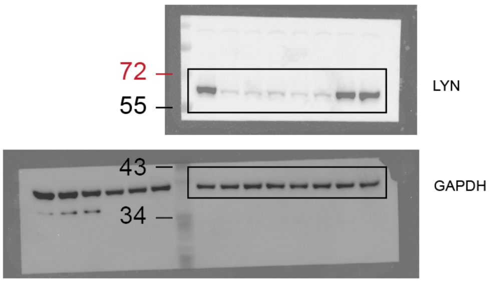

Replicate 3 (annotated blot)

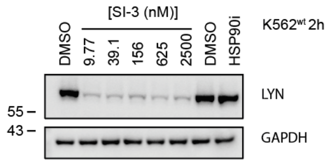

Uncropped immunoblots and further replicates to Fig 2c.

Replicate 1 (original)

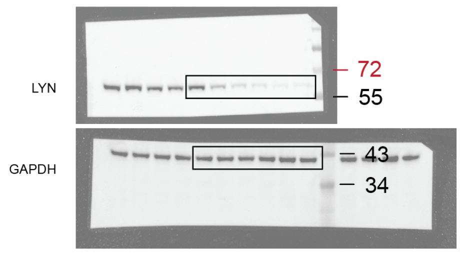

Replicate 2 (original)

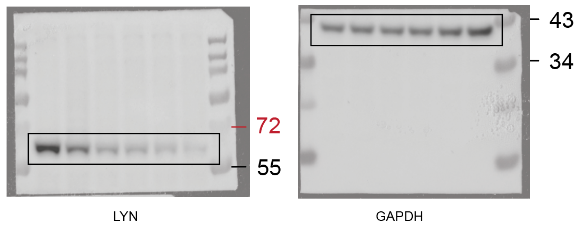

Replicate 2 (annotated blot)

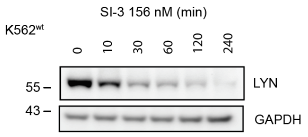

Replicate 3 (annotated blot)

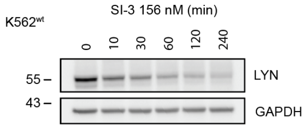

Replicate 3 (original)

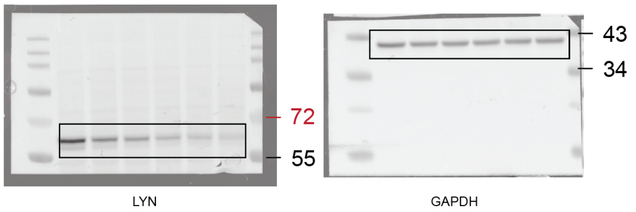

Uncropped immunoblots and further replicates to Fig 2d.

Replicate 1 (original)

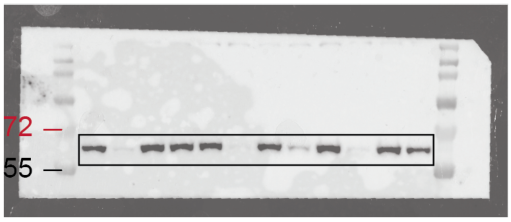

LYN

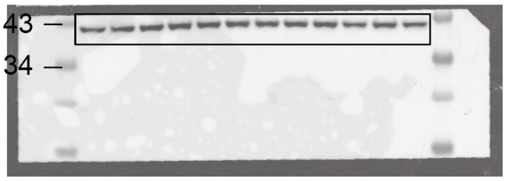

GAPDH

Replicate 2 (annotated)

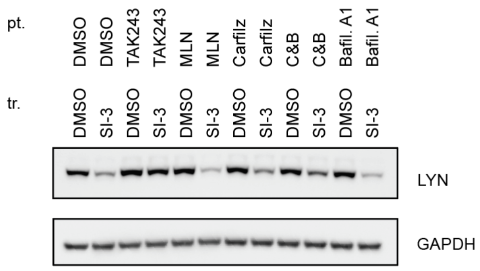

Replicate 2 (original)

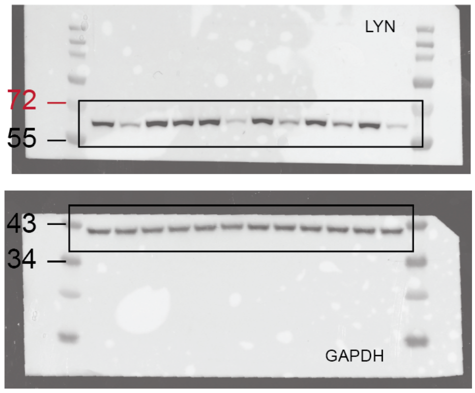

Replicate 3 (annotated)

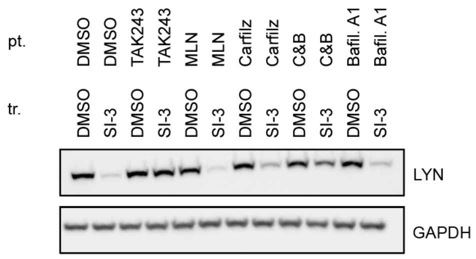

Replicate 3 (original)

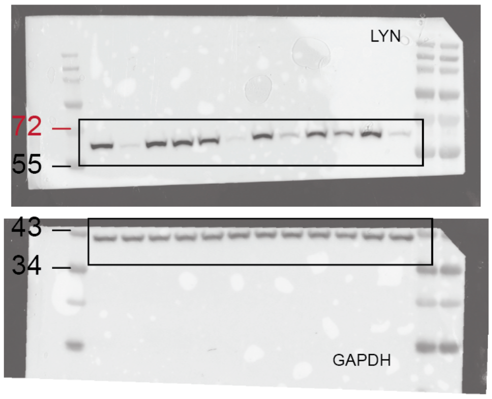

Uncropped immunoblots and further replicates to Fig 3d.

Replicate 1 (original)

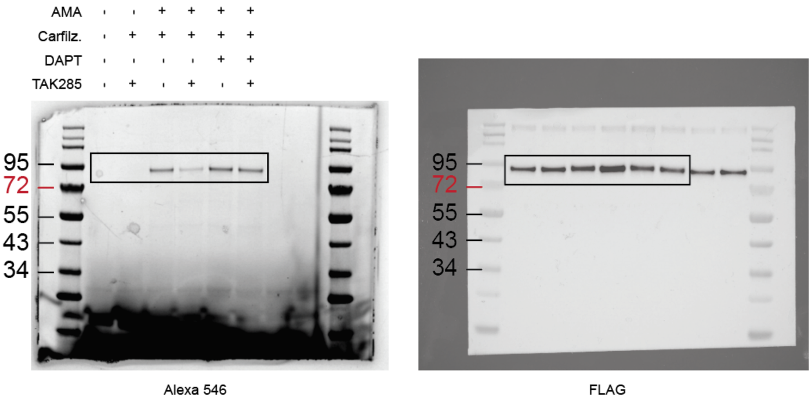

Replicate 2 (original)

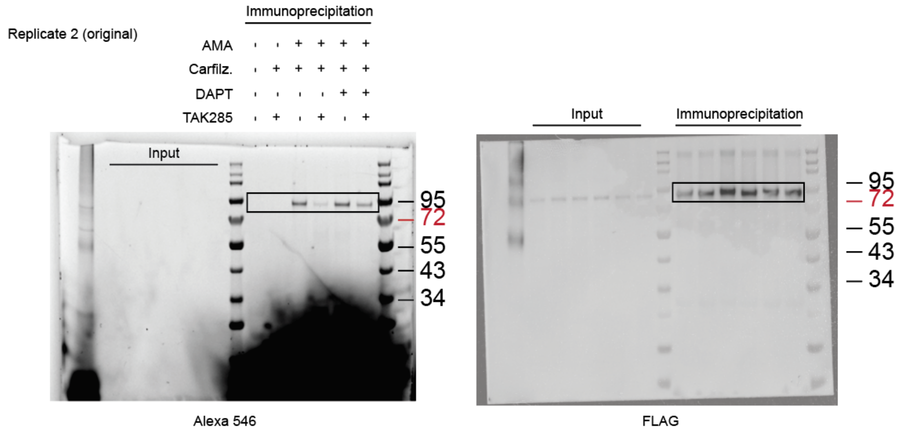

Replicate 3 (original)

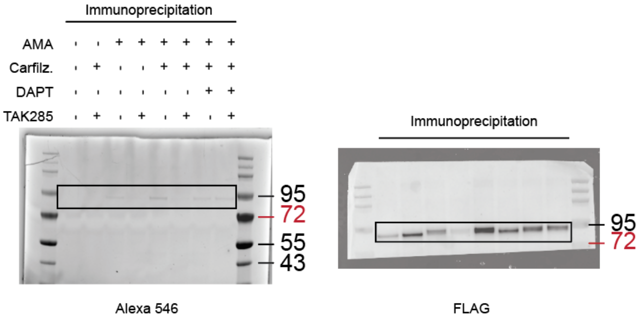

Uncropped immunoblots and further replicates to Fig 4c.

Replicate 1 (original)

RIPK2

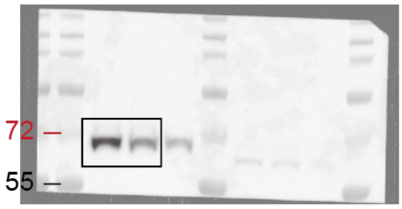

GAPDH

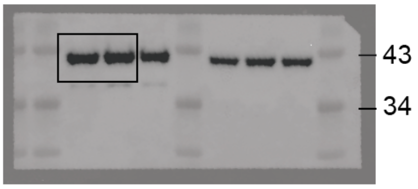

Replicate 2 (annotated)

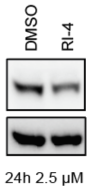

Replicate 2 (original)

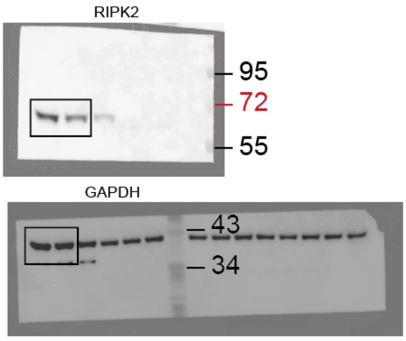

Replicate 3 (annotated)

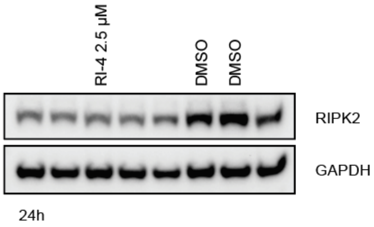

Replicate 3 (original)

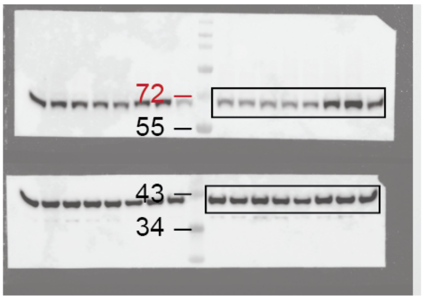

Uncropped immunoblots and further replicates to Fig 4f.

Replicate 1 (original)

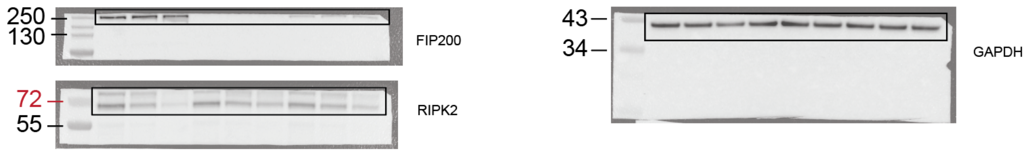

Replicate 2 (annotated)

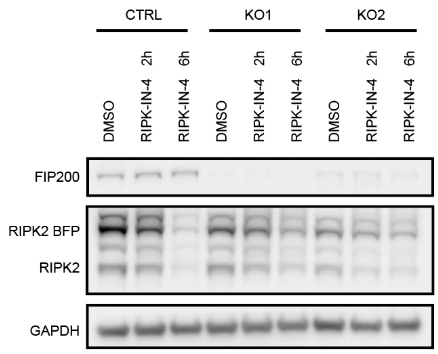

Replicate 2 (original)

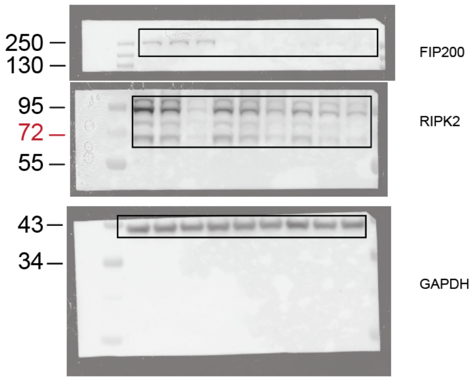

Replicate 3 (annotated)

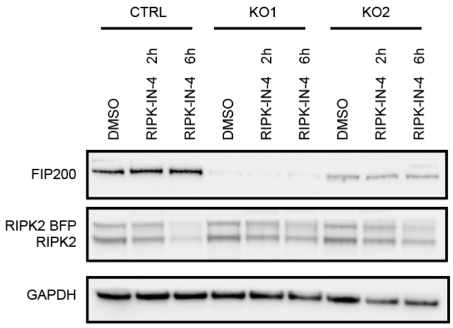

Replicate 3 (original)

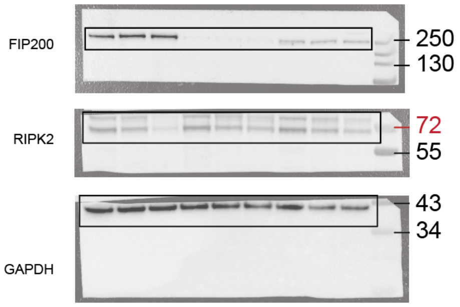

Uncropped immunoblots to Fig 4l.

Original

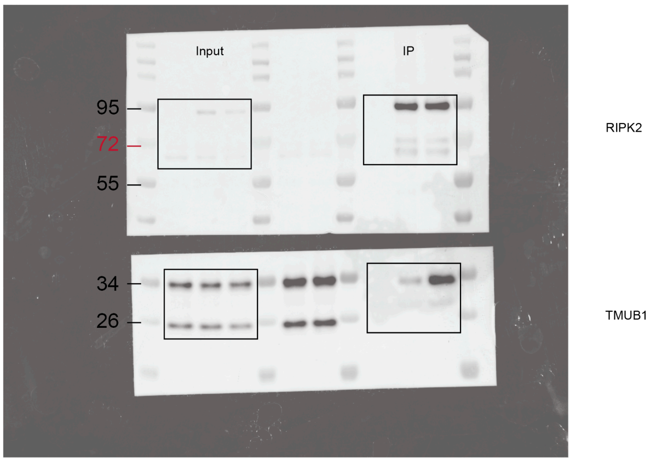

Uncropped immunoblots and further replicates to Extended Data Fig 1d.

Replicate 1 (original)

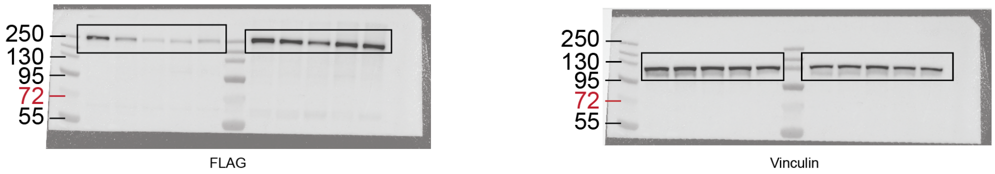

Replicate 2 (annotated)

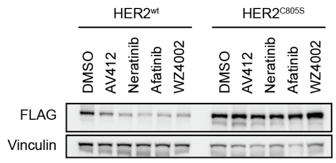

Replicate 2 (original)

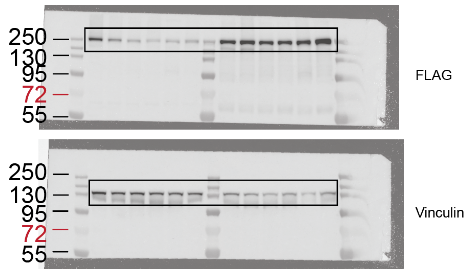

Replicate 3 (annotated)

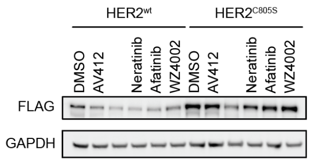

Replicate 3 (original)

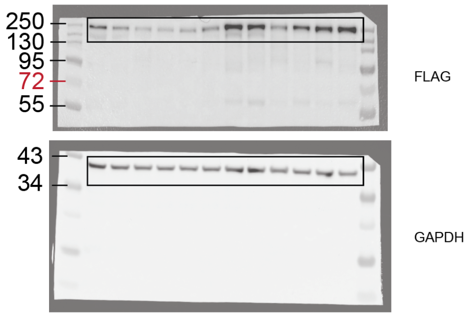

Uncropped immunoblots and further replicates to Extended Data Fig 4e.

LYN

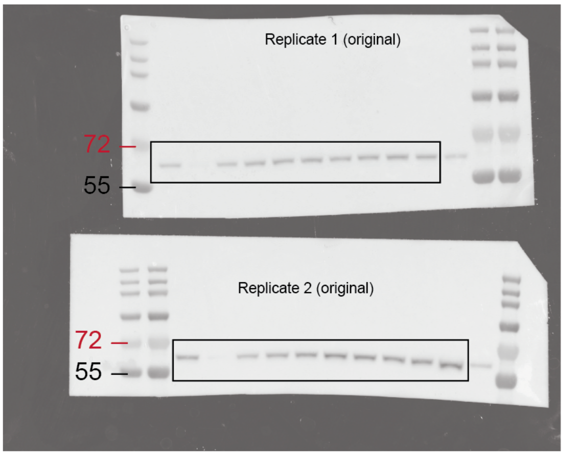

GAPDH

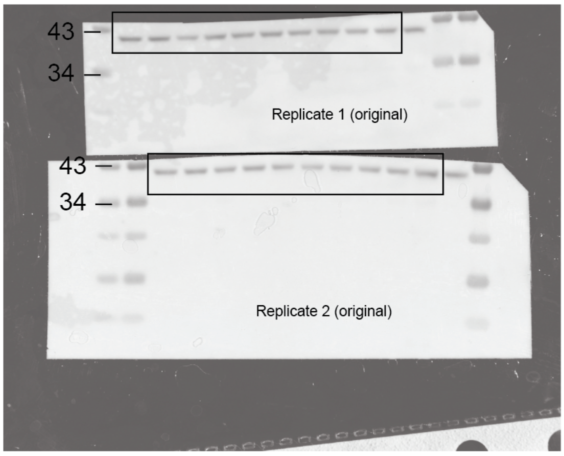

Replicate 3 (original)

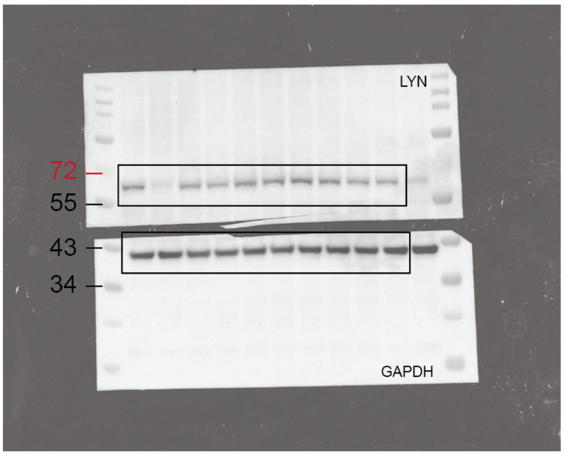

Replicate 2 (annotated)

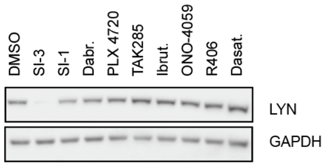

Replicate 3 (annotated)

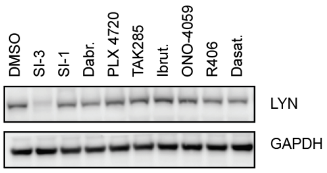

Uncropped immunoblots and further replicates to Extended Data Fig 4h.

Replicate 1 (original)

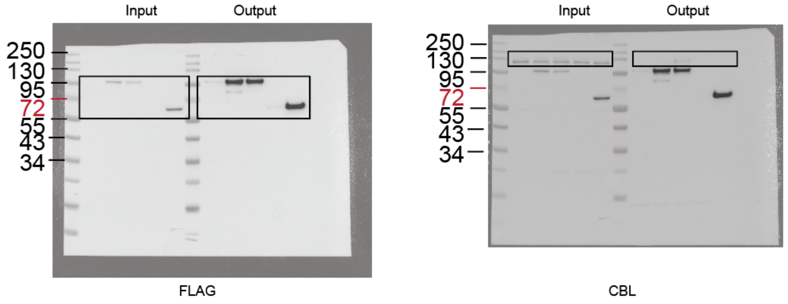

Replicate 2 (annotated)

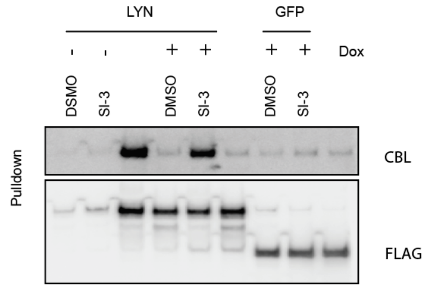

Replicate 2 (original)

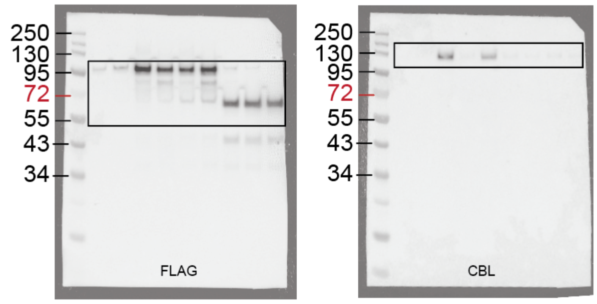

Replicate 3 (annotated)

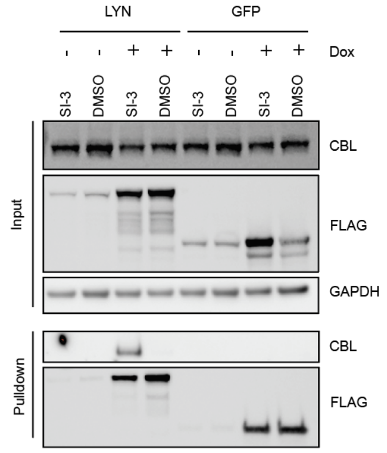

Replicate 3 (original)

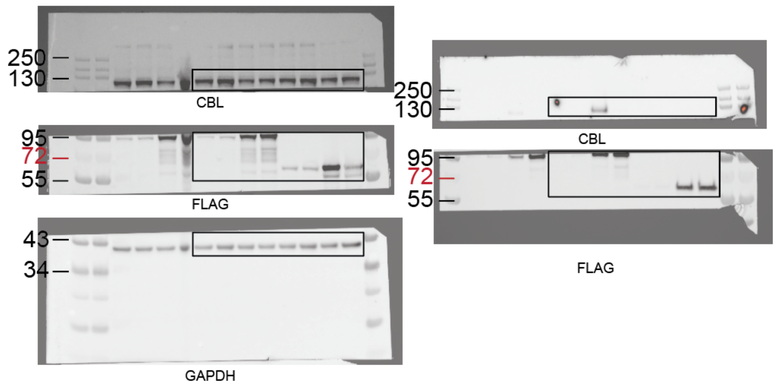

Uncropped immunoblots and further replicates to Extended Data Fig 4j.

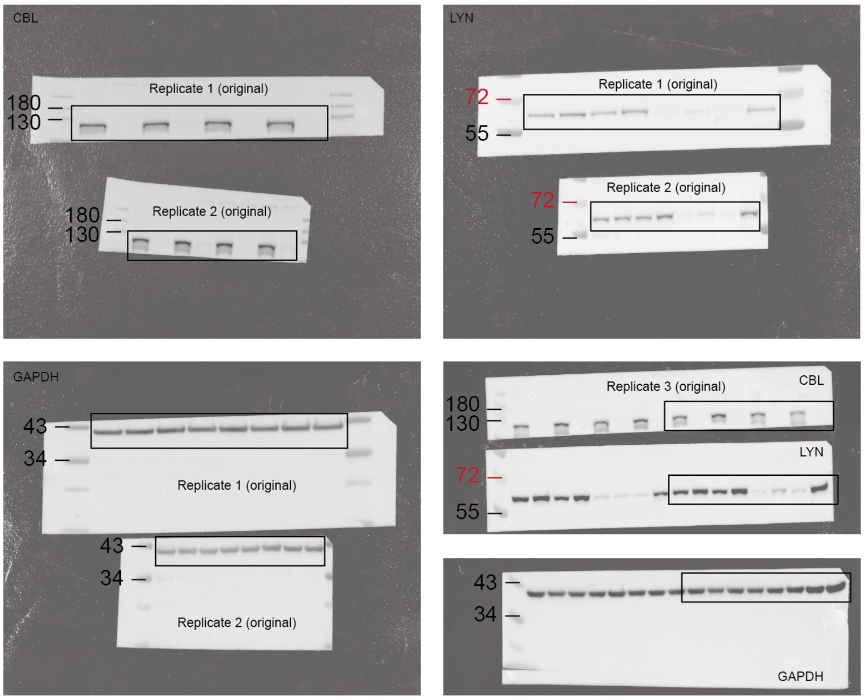

Replicate 2 (annotated)

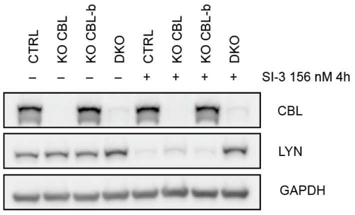

KBM7 iCas9

Replicate 3 (annotated)

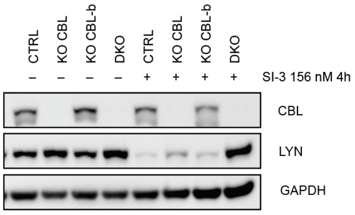

KBM7 iCas9

Uncropped immunoblots and further replicates to Extended Data Fig 5b.

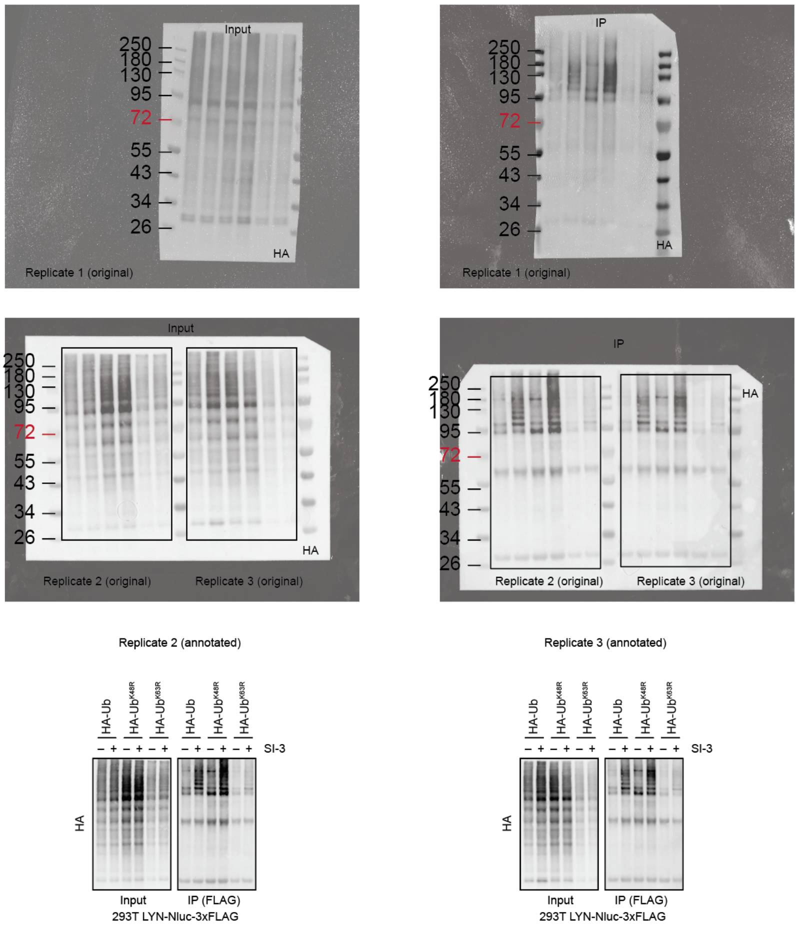

Uncropped immunoblots and further replicates to Extended Data Fig 5g.

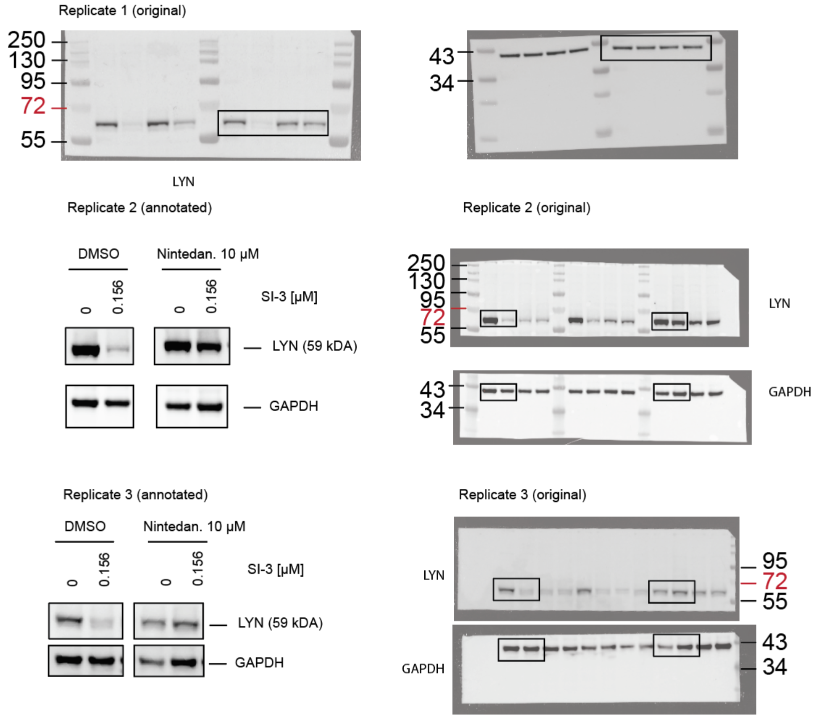

Uncropped immunoblots and further replicates to Extended Data Fig 5h.

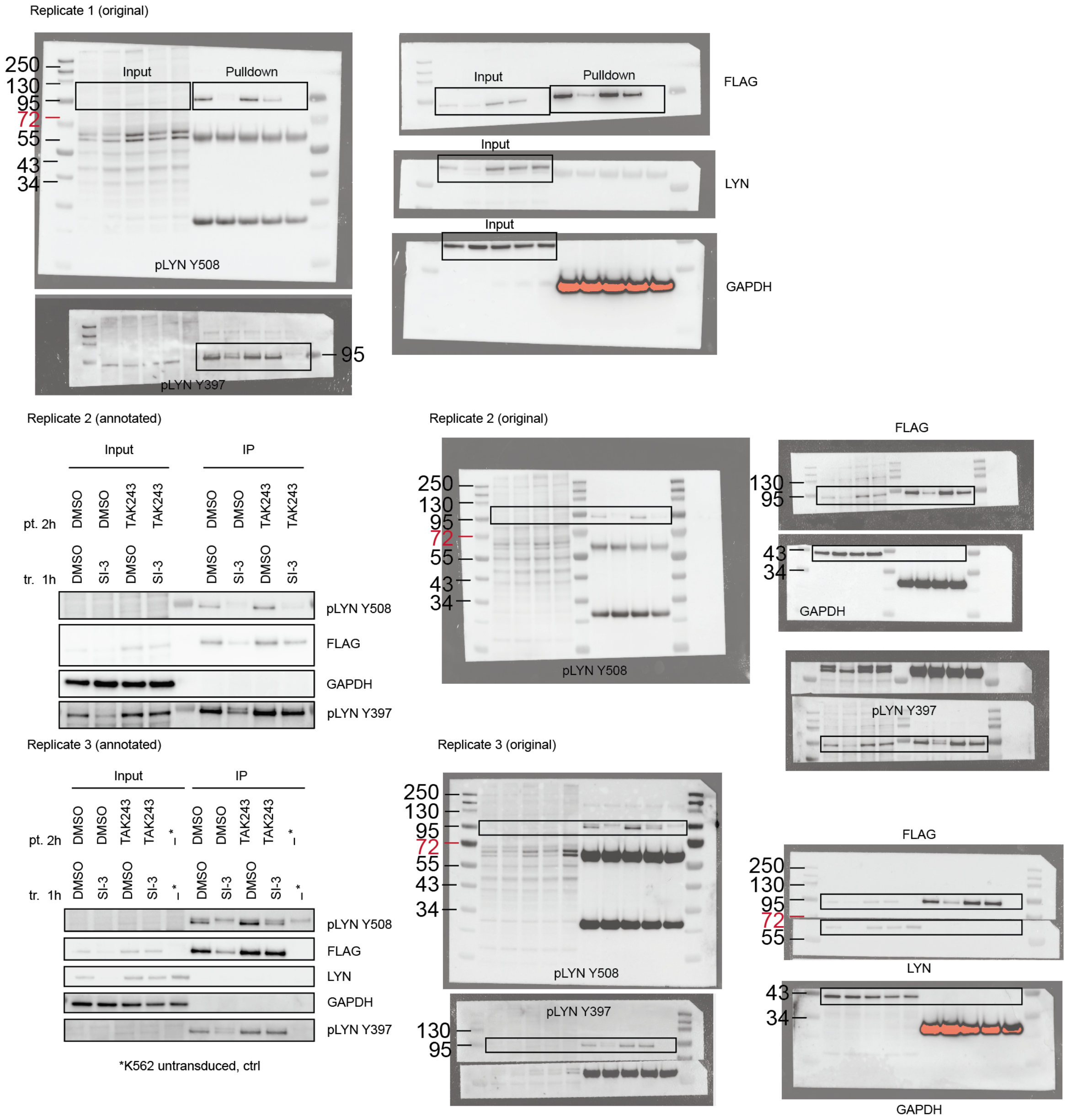

Uncropped immunoblots and further replicates to Extended Data Fig 6f.

Replicate 1 (original)

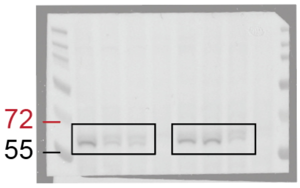

BLK

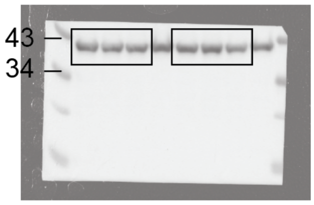

GAPDH

Replicate 2 (annotated)

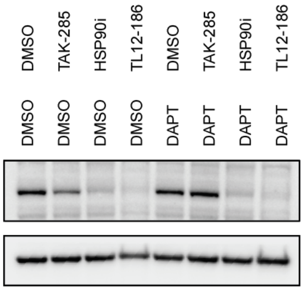

Replicate 2 (original)

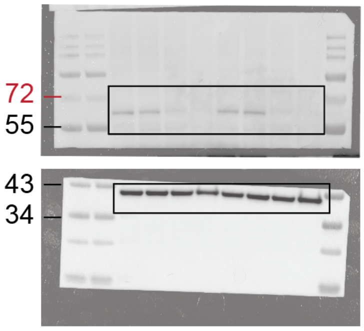

Replicate 3 (annotated)

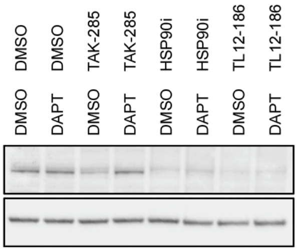

Replicate 3 (original)

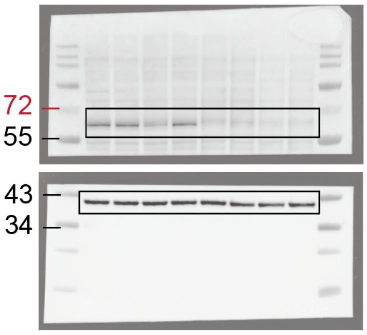

Uncropped immunoblot to Extended Data Fig 7i.

Originals

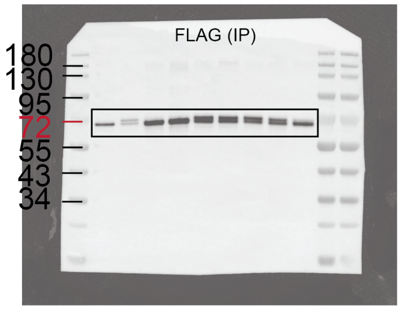

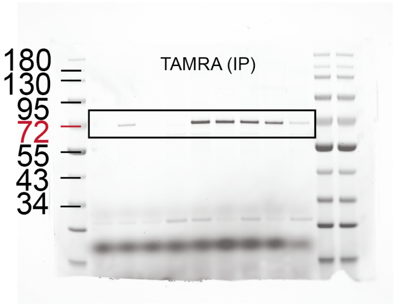

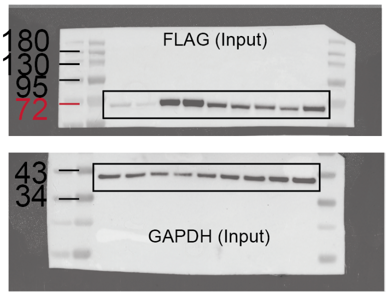

Uncropped immunoblots and further replicates to Extended Data Fig 8e.

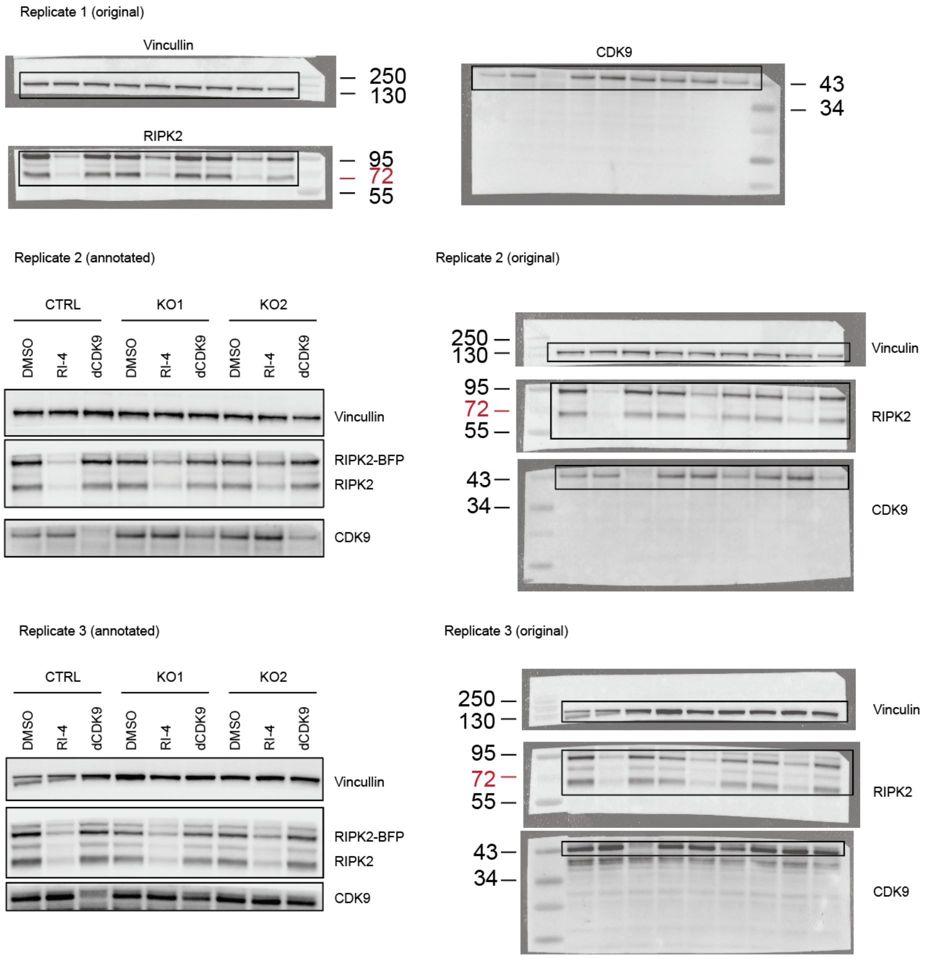

Gating strategy: iCas9 GFP with Thy1.1 Neo sgRNAs & sorted/unsorted reporter examples

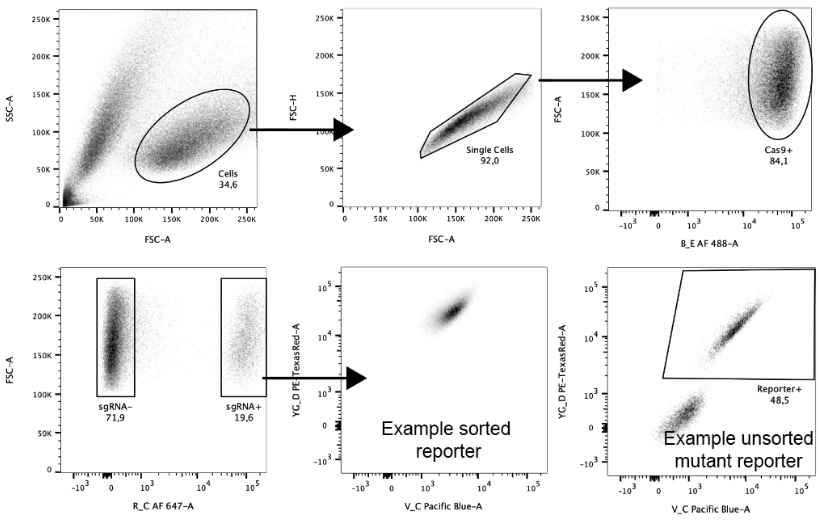

Gating strategy: CRISPR screen

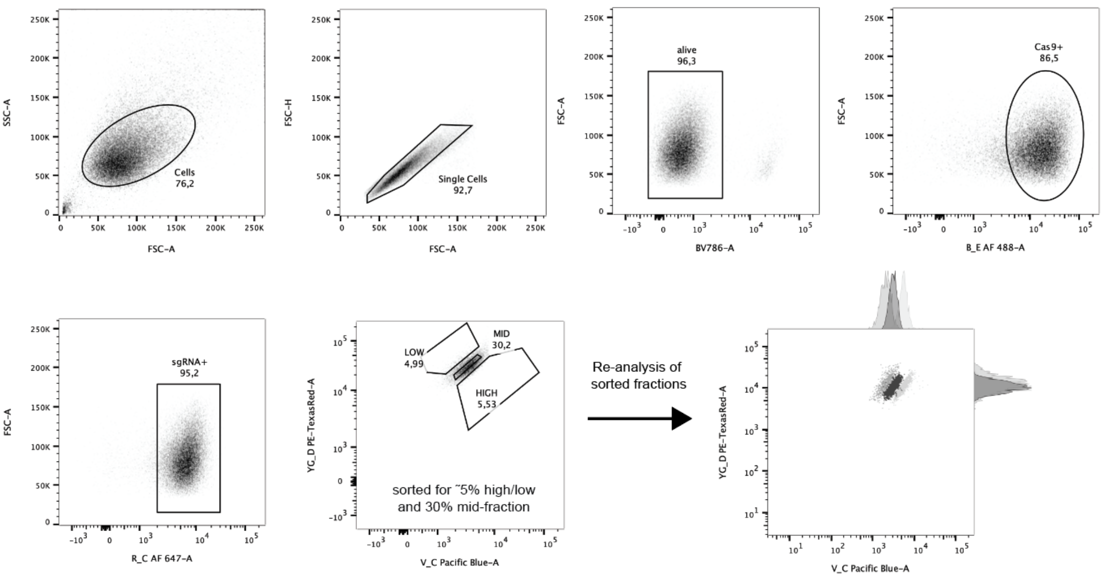

Supplementary Fig S2. Gating strategies applied across Fig 2-4 and Extended Data Fig 3-9. Top: Hierarchy of applied gating scheme for either sorted reporters (pool and clonal or unsorted cell lines. iCas9+ (AF488/GFP) and Thy1.1+ (AF647) gating was skipped in case of non-induced Cas9 (non-induced k.o. experiments), and/or in case cells had been selected for harboring the sgRNA using Neomycin. **Bottom:** Gating strategy applied for all CRISPR/Cas9 screens and exemplified re-analysis of the sorted fraction after FACS.

Supplementary Fig S3. NMR and HPLC-MS (ESI+) for synthesis of amine-tethered TAK285

^{1}H-NMR spectrum of *tert*-butyl (2-bromoethyl)carbamate

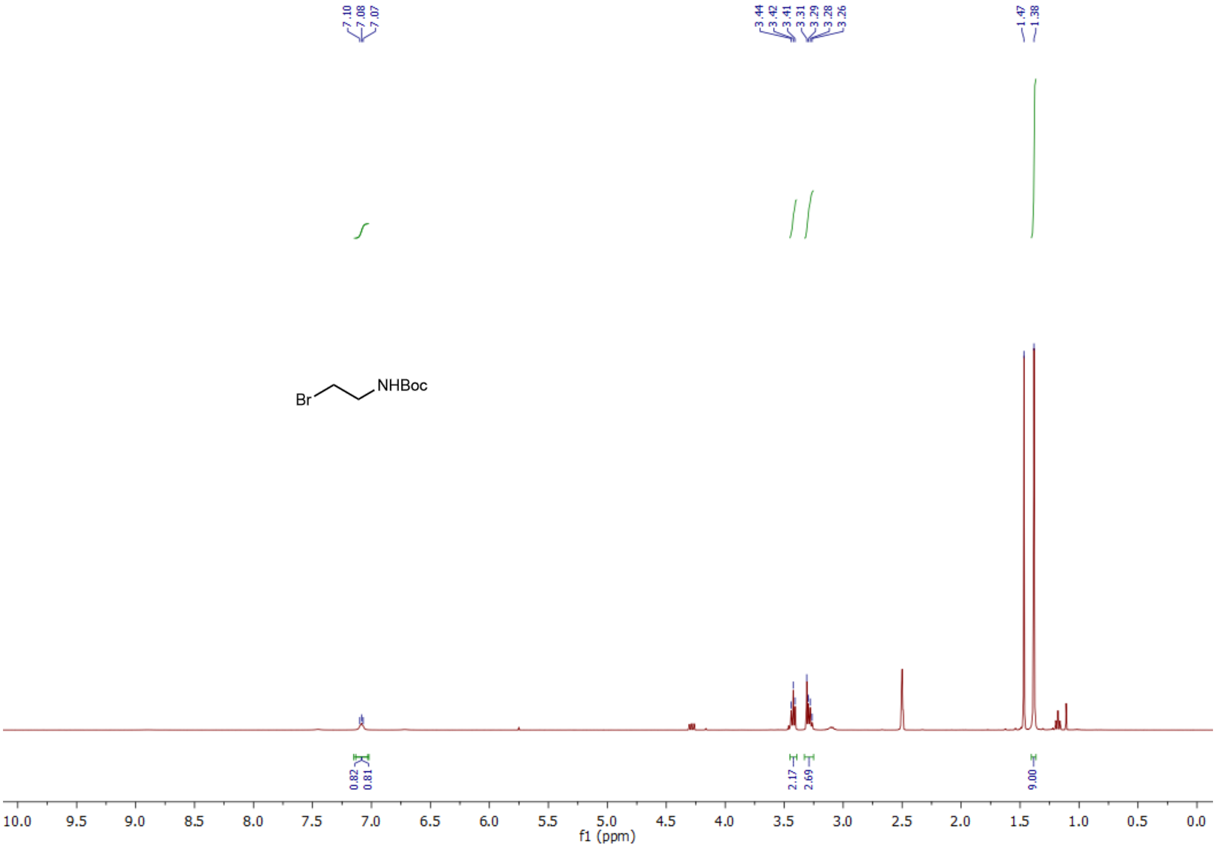

^{1}H-NMR spectrum of *tert*-butyl (2-(4-chloro-5*H*-pyrrolo[3,2-*d*]pyrimidin-5-yl)ethyl)carbamate

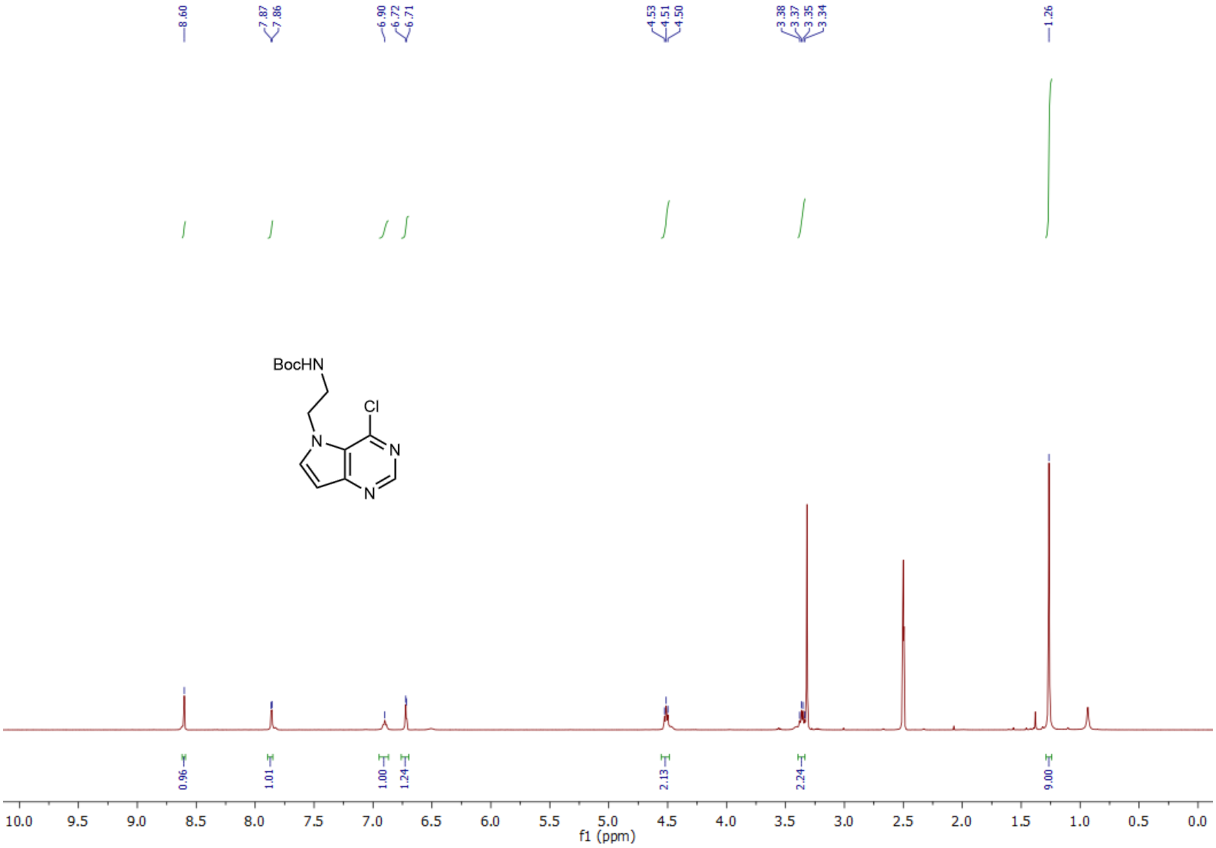

^{1}H-NMR spectrum of 2-chloro-4-nitro-1-(3-(trifluoromethyl)phenoxy)benzene

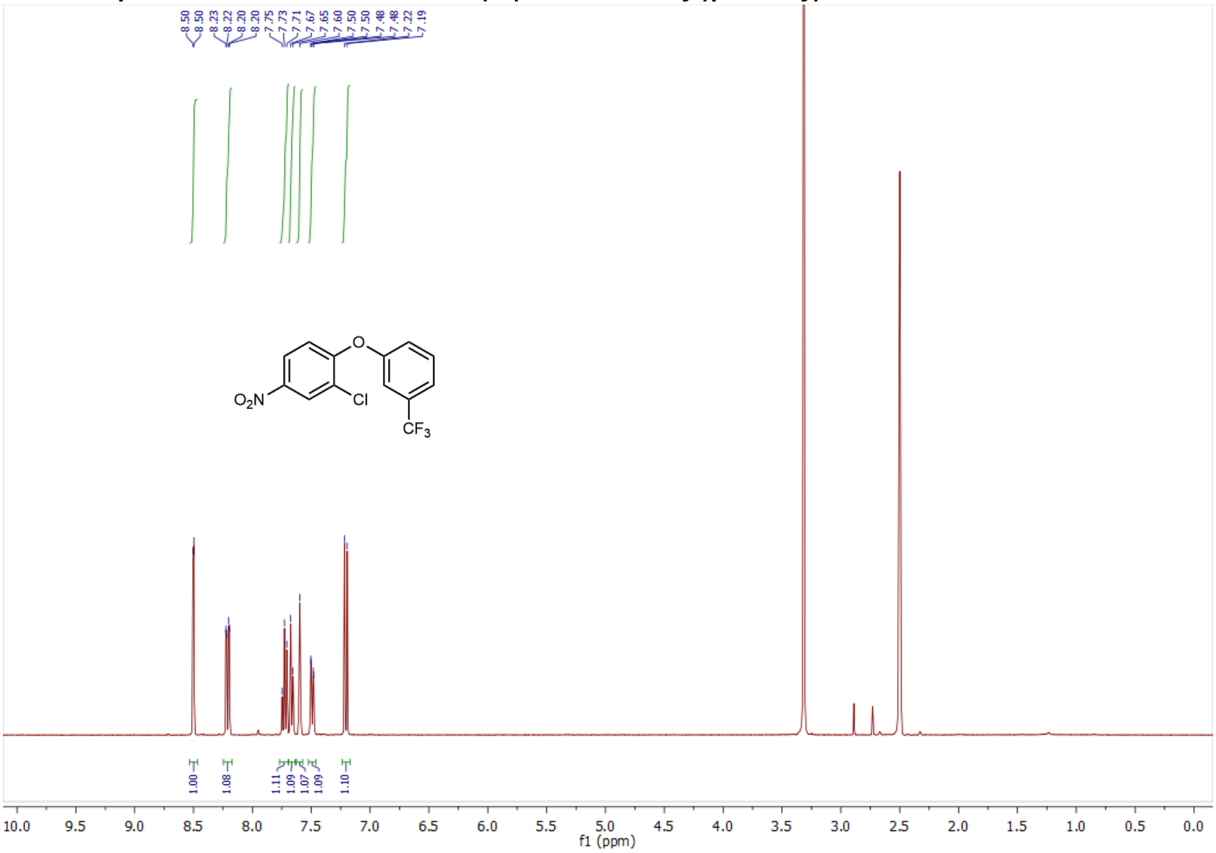

^{1}H-NMR spectrum of 3-chloro-4-(3-(trifluoromethyl)phenoxy)aniline

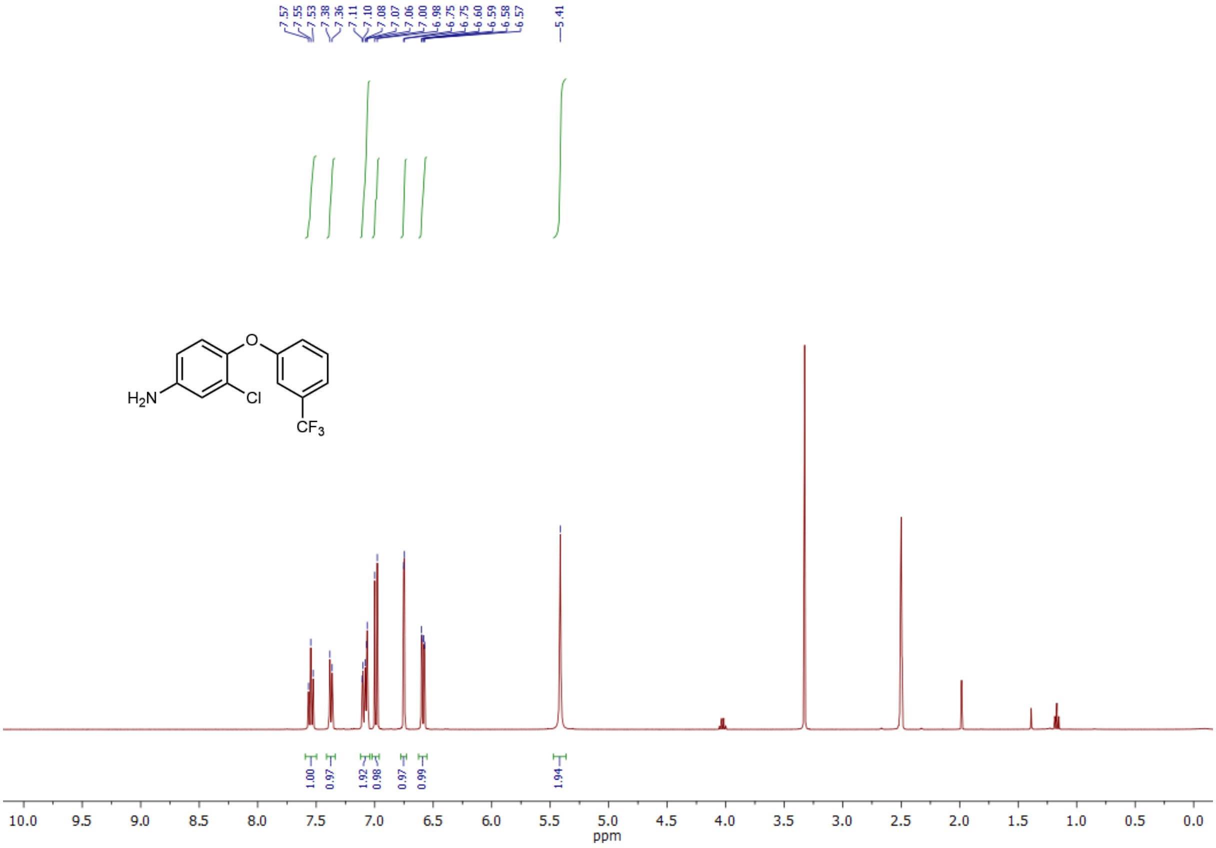

^{1}H-NMR spectrum of 5-(2-aminoethyl)-*N*-(3-chloro-4-(3-(trifluoromethyl)phenoxy)phenyl)-5*H*-pyrrolo[3,2-*d*]-pyrimidin-4-amine

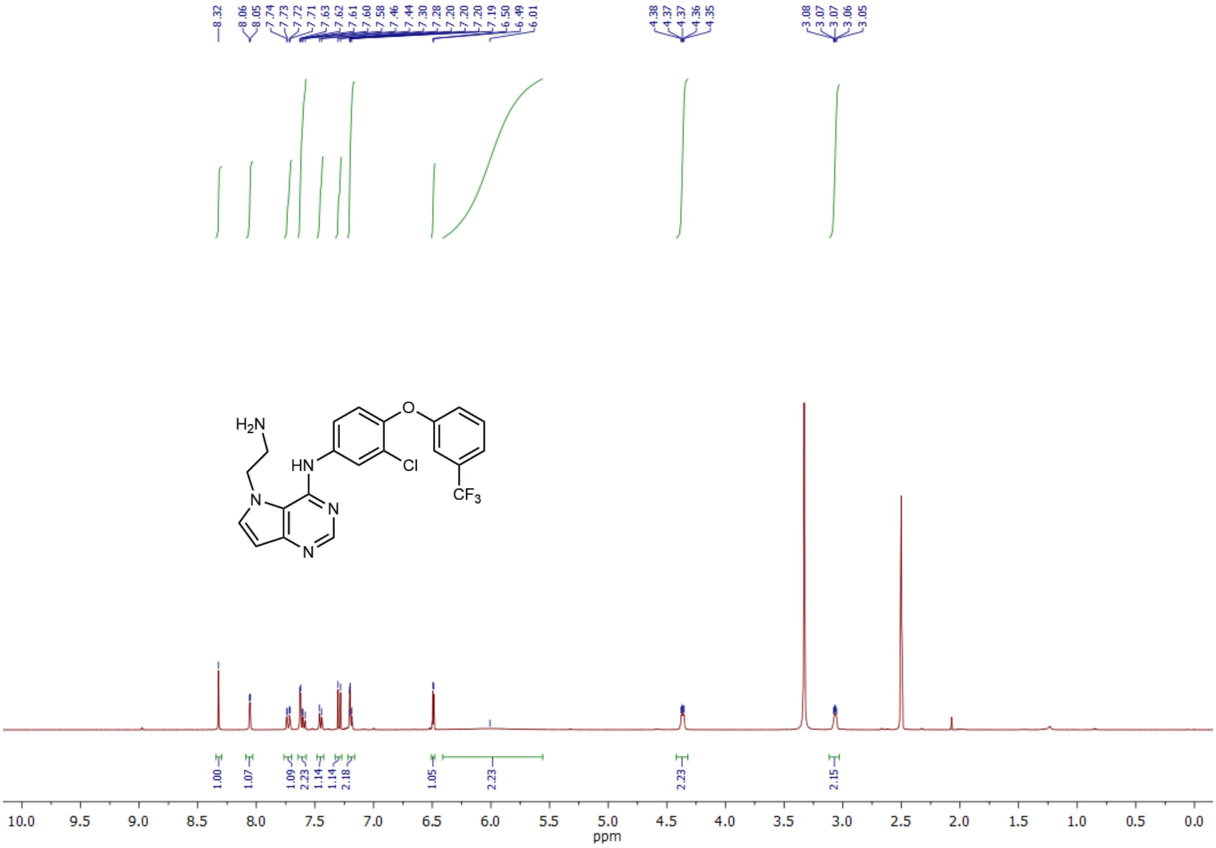

^{1}H-NMR spectrum of benzyl (6-((2-(4-((3-chloro-4-(3-(trifluoromethyl)phenoxy)phenyl)amino)-5*H*-pyrrolo-[3,2-*d*]pyrimidin-5-yl)ethyl)amino)hexyl)carbamate

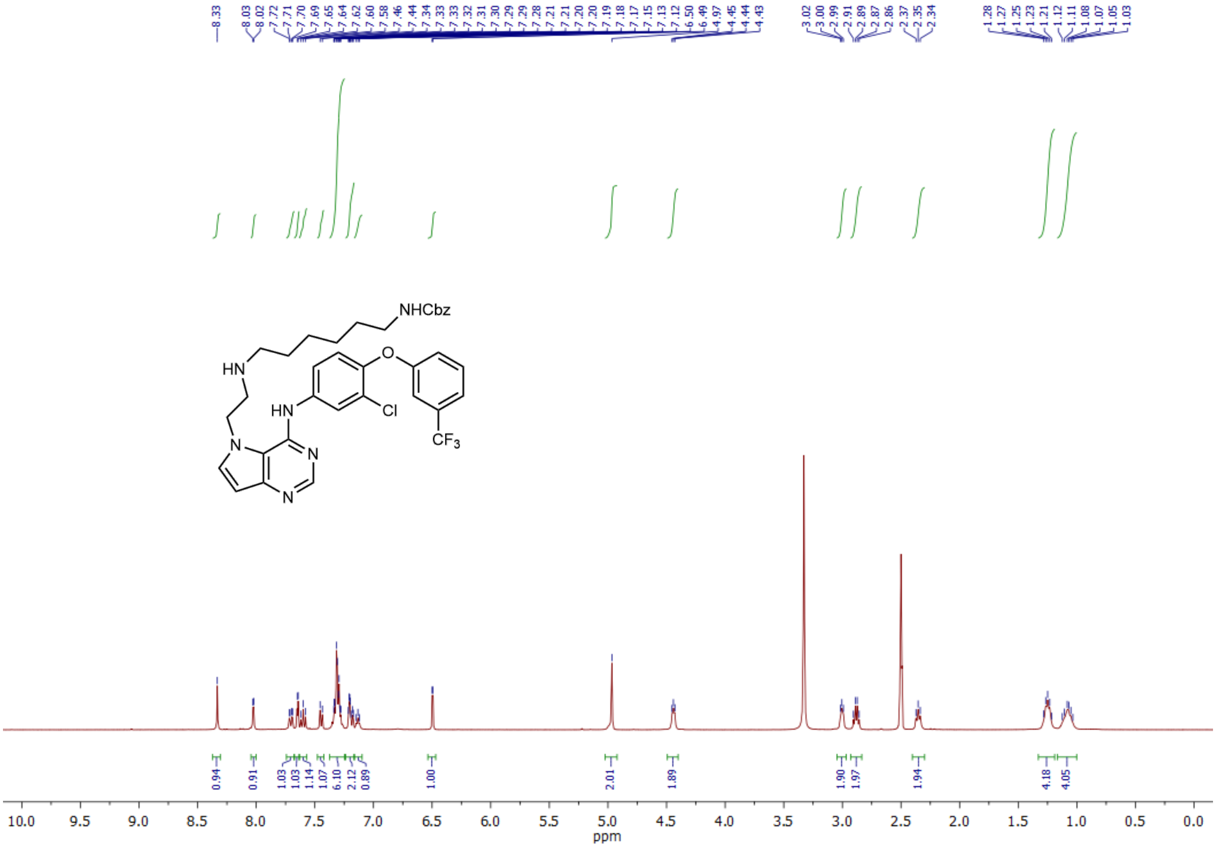

^{1}H-NMR spectrum of benzyl (6-(*N*-(2-(4-((3-chloro-4-(3-(trifluoromethyl)phenoxy)phenyl) amino)-5*H*-pyrrolo-[3,2-*d*]pyrimidin-5-yl)ethyl)-3-hydroxy-3-methylbutanamido)hexyl)carbamate

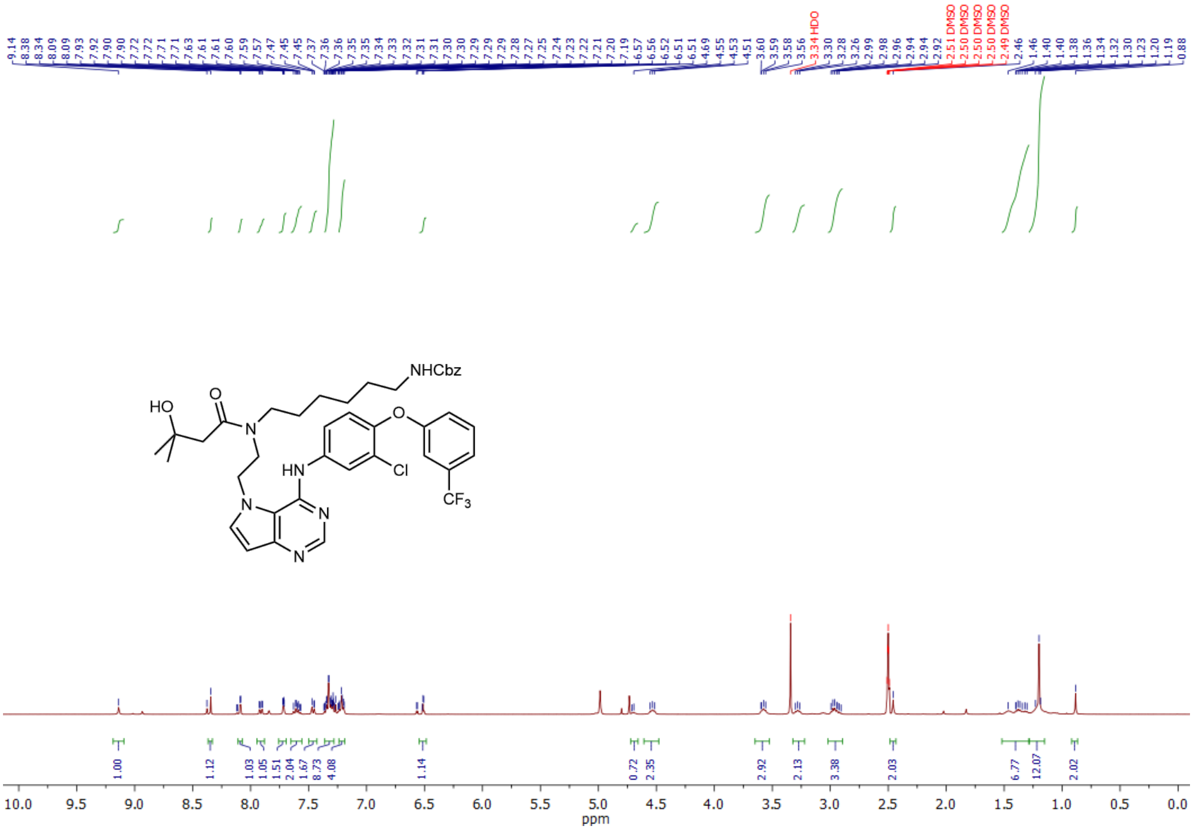

^{1}H-NMR spectrum of *N*-(6-aminohexyl)-*N*-(2-(4-((3-chloro-4-(3-(trifluoromethyl)phenoxy)-phenyl)-amino)-5*H*-pyrrolo[3,2-*d*]pyrimidin-5-yl)ethyl)-3-hydroxy-3-methylbutanamide

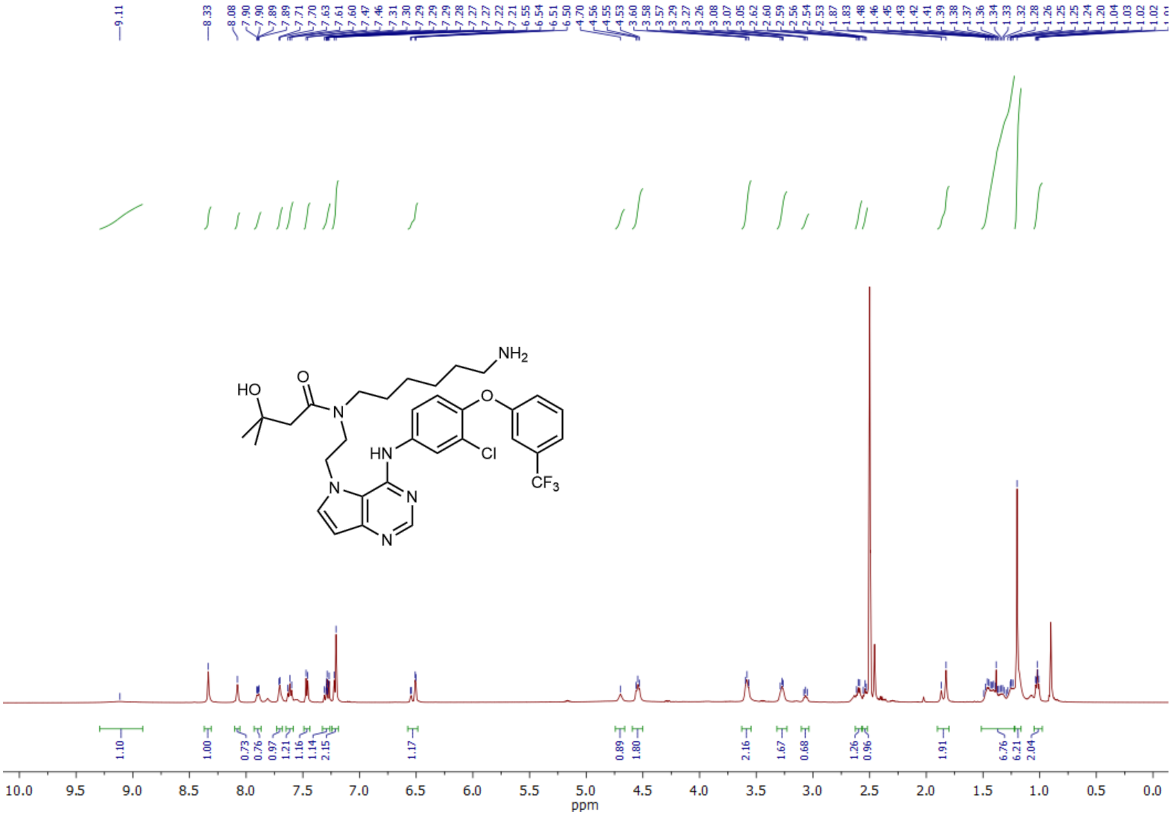

^{13}C-NMR spectrum of *N*-(6-aminohexyl)-*N*-(2-(4-((3-chloro-4-(3-(trifluoromethyl)phenoxy)-phenyl)-amino)-5*H*-pyrrolo[3,2-*d*]pyrimidin-5-yl)ethyl)-3-hydroxy-3-methylbutanamide

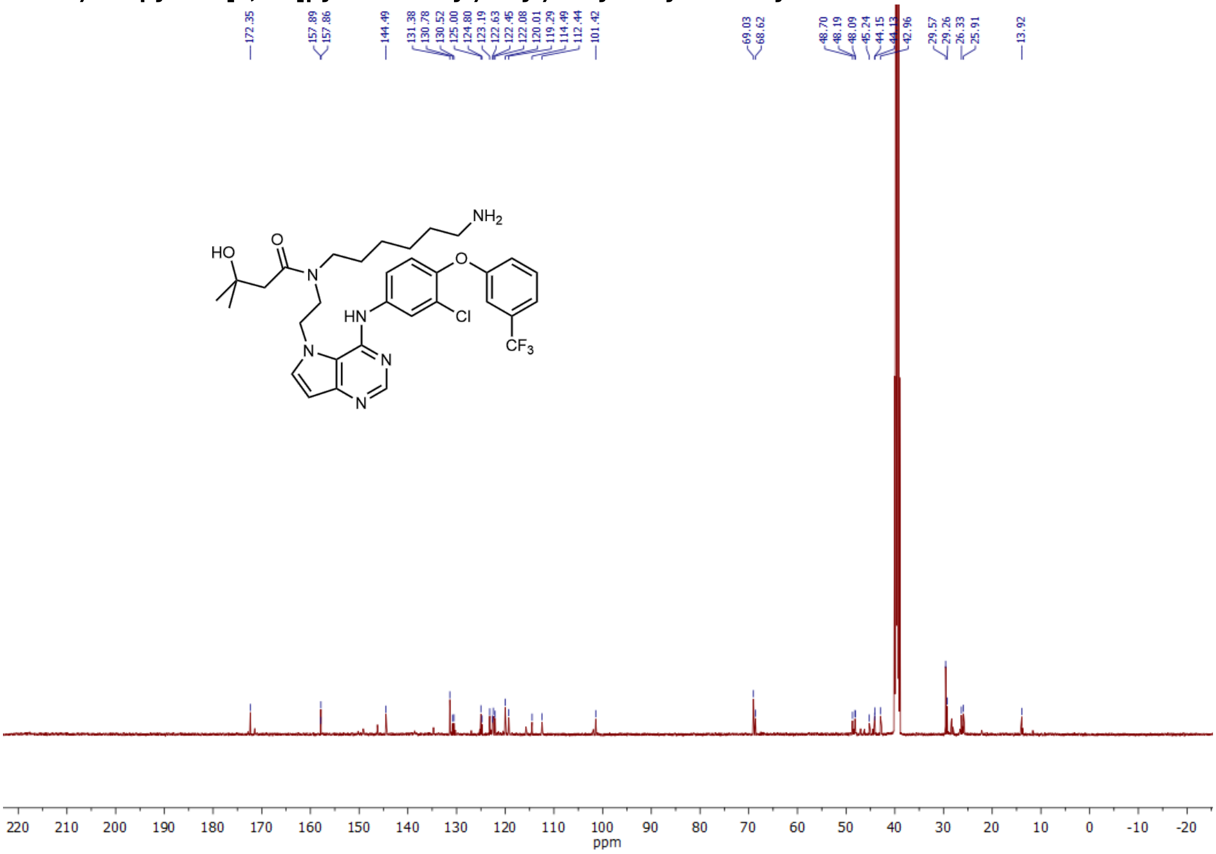

ESI+-HRMS of *N*-(6-aminohexyl)-*N*-(2-(4-((3-chloro-4-(3-(trifluoromethyl)phenoxy)-phenyl)-amino)-5*H*-pyrrolo[3,2-*d*]pyrimidin-5-yl)ethyl)-3-hydroxy-3-methylbutanamide

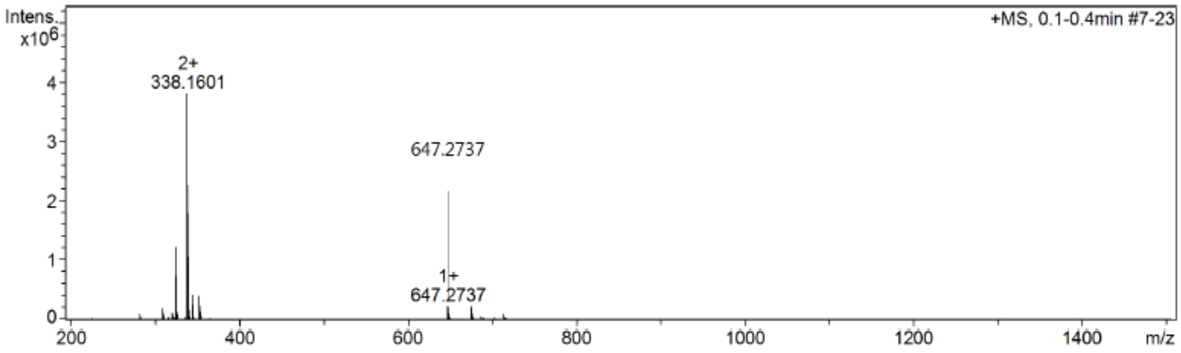

HPLC purity control of *N*-(6-aminohexyl)-*N*-(2-(4-((3-chloro-4-(3-(trifluoromethyl)phenoxy)-phenyl)-amino)-5*H*-pyrrolo[3,2-*d*]pyrimidin-5-yl)ethyl)-3-hydroxy-3-methylbutanamide

Max Area%	98.266
UV Signal Purity>95%	Pass

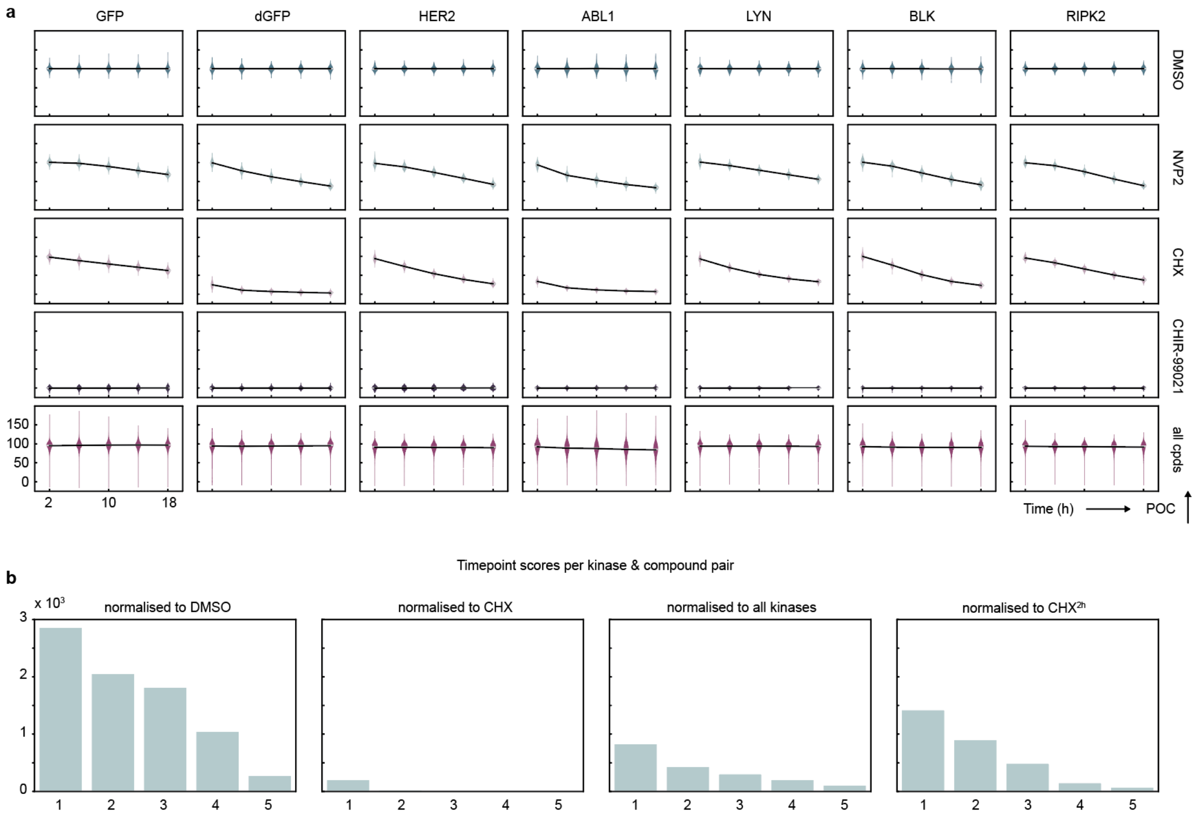

Supplementary Fig S4. Normalisation and overview of scoring distribution. a Selected examples for kinases and controls highlighted throughout the paper. Depiction of the screening trajectories as line (black) and violin plot (kernel density estimate (KDE) for data including a horizontal grey line highlighting the median) for all the controls and averaged across the screened compounds (all cpds, not including the PROTAC controls). DMSO is shown in green, NVP2 in light green, CHX (cycloheximide) in light pink, CHIR-99021 in dark lilac and the average of all cpds in pink. DMSO and CHIR-99021 formed the basis of the data normalisation to generate the POC values. CHX trajectories were used for determining each reporter's half-life. **b** Score overview of each kinase and compound pair across the different time points for each chosen normalisation strategy. A score of 5 indicates the kinase-compound trajectory scored for each recorded timepoint, a score of 1 indicates scoring for a single timepoint across the recorded trajectory. Recorded time stamps: 2,6,10,14 and 18h.

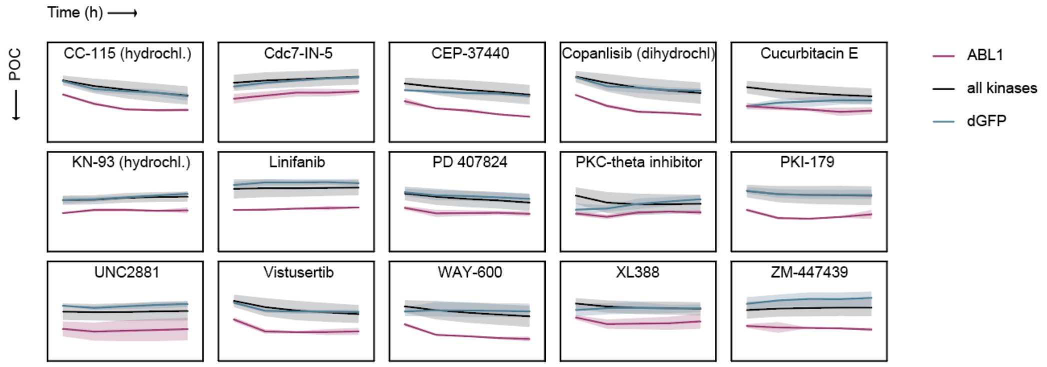

Supplementary Fig S5. Continuation from Extended Data Fig 1g. Drug screening data comparing ABL1 to the mean of all other kinases and the dGFP control trajectory (m = 2, error bars correspond to the CI for individual trajectories and the SD for the mean of all kinases).

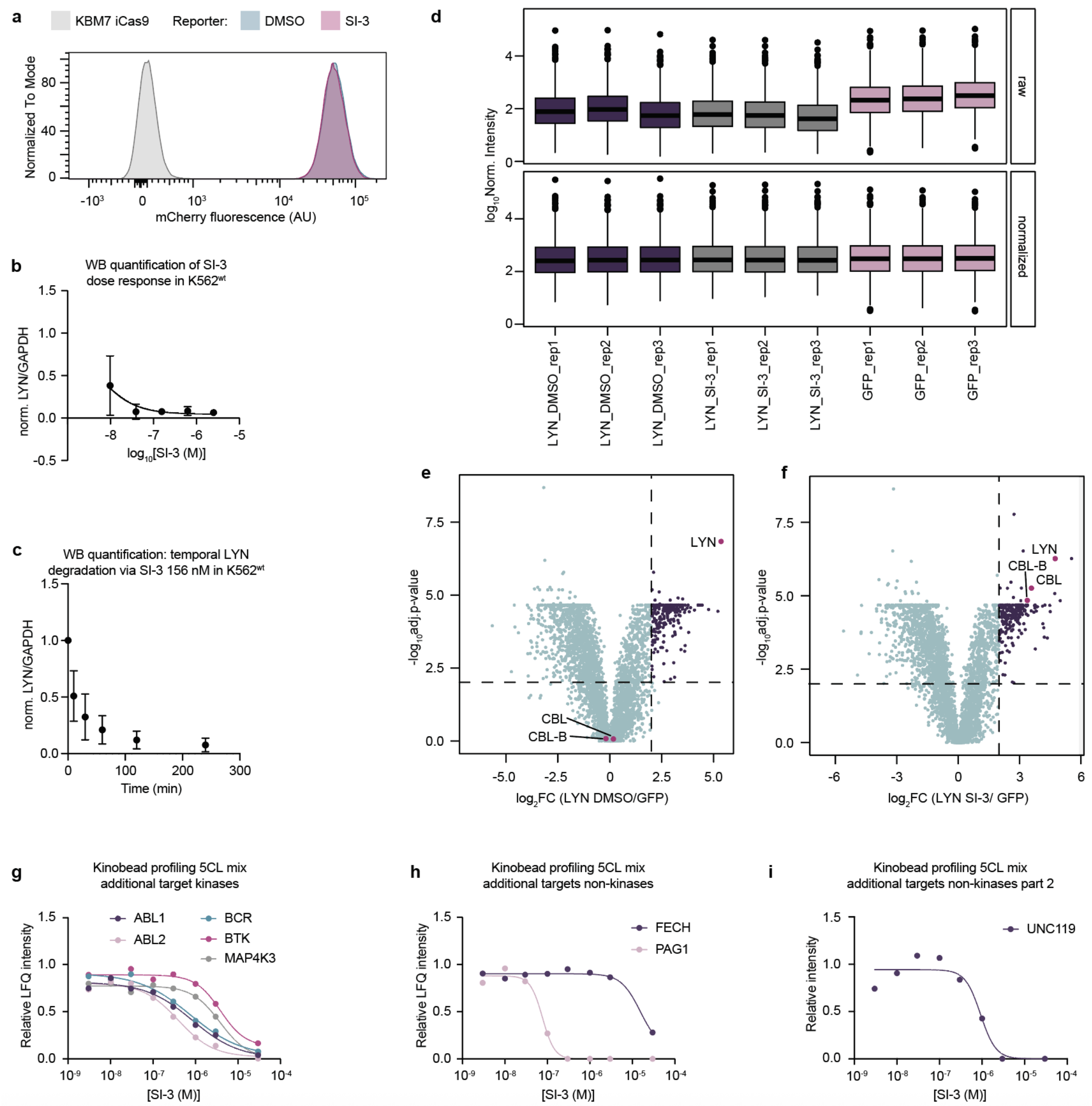

Supplementary Fig S6. Additional data to LYN datasets. a Flow histogram matched to **Fig 2a** showing no change of mCherry fluorescence upon SI-3 treatment (8h, 156 nM). **b** Quantification of endogenous dose response measured via immunoblot at 2h after treatment of SI-3 with varying concentrations (9.77 nM – 2.5 µM, see example replicate in **Fig 2b**). Black line indicates the nonlinear fit of a dose response (inhibitor vs response with three parameters). D_{max} = 95.55%, IC50 = 4.595 nM, R^2 = 0.8767. Data is shown relative to the GAPDH loading control and normalised to DMSO (n = 3). **c** Quantification of data shown in **Fig 2c** and respective replicates monitoring endogenous LYN degradation for 156 nM SI-3 treatment over time by immunoblotting. Data is shown relative to the GAPDH loading control and normalised to DMSO (n = 3). **d** Distribution of protein intensities ($\log_{10}$) before and after total ion current (TIC) normalisation across LYN, LYN SI-3 treated and GFP negative controls. Solid line represents the median, box limits show the IQR and its whiskers 1.5 x IQR. Outliers are visualized as black dots (n = 3). **e** Differential enrichment of proteins in LYN DMSO control against GFP negative controls. The x-axis represents the $\log_2$FC of each protein against the signal in the GFP negative control. The y-axis shows the -$\log_{10}$ of the adjusted p-value (BH correction). As thresholds for significantly enriched proteins a $\log_2$FC of larger or equal to 2 and an adjusted p-value smaller or equal to 0.01 were used (dotted lines). Not significantly enriched proteins are shown in light green, whereas

significantly enriched proteins are indicated in purple. The LYN kinase bait protein, and the two E3-ligases CBL and CBL-B are shown in pink (n = 3). **f** Differential enrichment of proteins in LYN SI-3 treated against GFP negative controls. The x-axis represents the log_2FC of each protein against the signal in the GFP negative control. The y-axis shows the $-log_{10}$ of the adjusted p-value (BH correction). As thresholds for significantly enriched proteins a log_2FC of larger or equal to 2 and an adjusted p-value smaller or equal to 0.01 were used (dotted lines). Not significantly enriched proteins are shown in light green, whereas significantly enriched proteins are indicated in purple. The LYN kinase bait protein, and the two E3-ligases CBL and CBL-B are highlighted in pink (n = 3). **g** Kinobead profiling for additional kinase examples for SI-3 in the standard 5-cell line lysate (5CL) mix showing the relative LFQ intensity (y-axis) against the drug concentration (x-axis). Following K_Ds were determined: ABL1 = 353 nM, ABL2 = 40.2 nM, BCR = 127 nM, BTK = 3.08 µM, MAP4K3 = 9.70 µM. **h** Plot as in G for for additional non-kinase examples Following K_Ds were determined: FECH = 6.35 µM, PAG1 = 48.4 nM **i** Plot as in (g) but modified to show the relative intensity (y-axis) for UNC119, K_D = 24.1 nM. n = biological replicates.

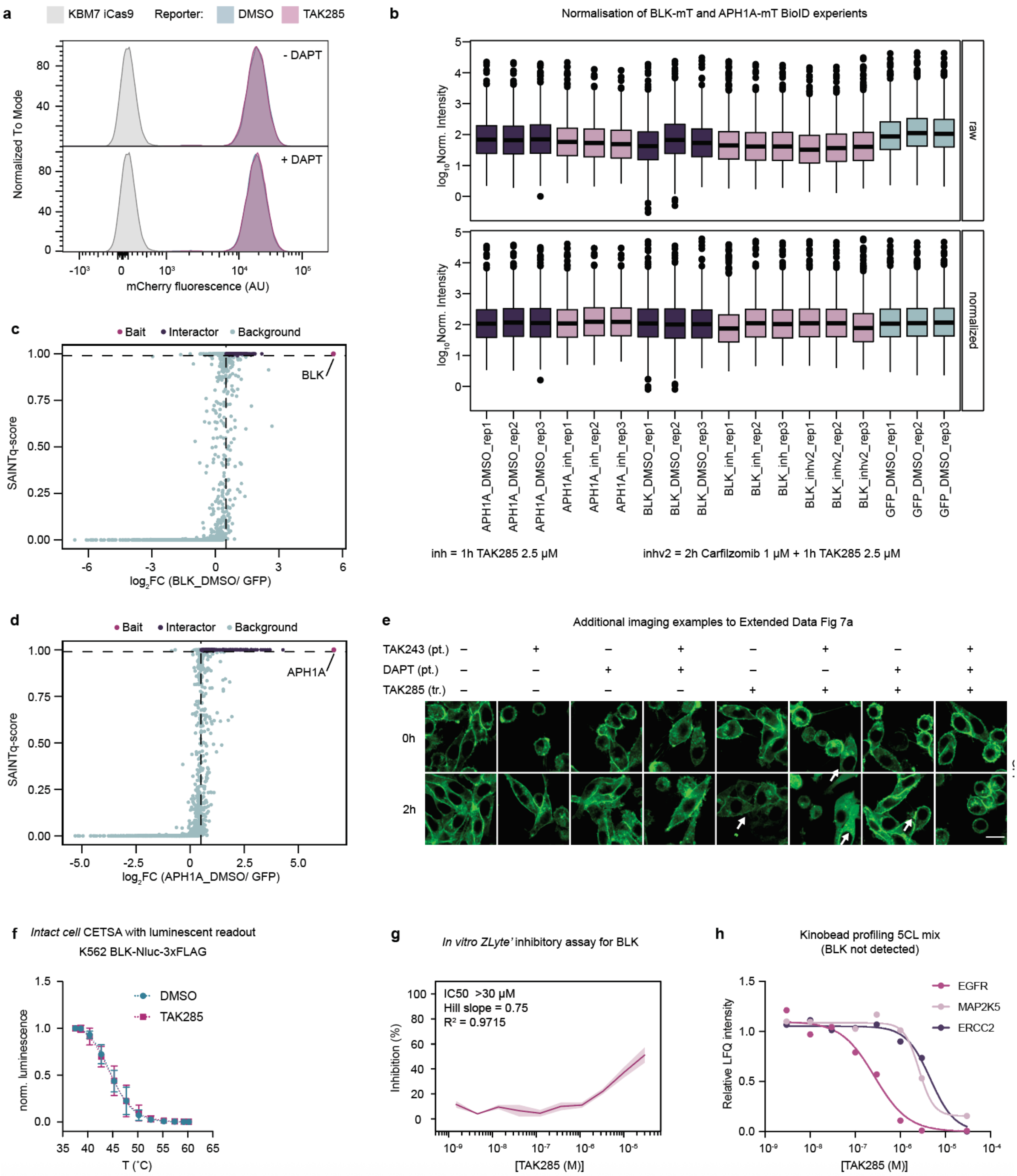

Supplementary Fig S7 Additional data to BLK datasets. **a** Flow histogram matched to **Extended Data Fig 6e** showing no change of mCherry fluorescence upon DAPT (pre-treated 2h, 12.5 μM) or TAK285 (6h, 2.5 μM) treatment. **b** Distribution of protein intensities (log_{10}) before and after total ion current (TIC) normalisation across BLK, APH1A and GFP negative controls including baseline (DMSO) and inhibitor treated conditions. Solid line represents the median, box limits show the IQR and its whiskers 1.5 x IQR. Outliers are visualized as black dots (n = 3). **c** Scatterplot of interaction partners after SAINTq scoring for BLK. The log_2FC against GFP negative controls (x-axis) was plotted against the SAINTq-score (y-axis). Proteins with a log_2FC of larger or equal to 0.5, a SAINTq score higher or equal to 0.99 with a BFDR smaller or equal to 0.01 and a CRAPome frequency below 10% were classified as interaction partners (pink). Proteins passing the scoring threshold but having a high CRAPome frequency were flagged together with the non-enriched proteins as background (light green) (n = 3). **d** Identical scatterplot of scoring characteristics for APH1A BioID experiments (n = 3). **e** Additional representative images of imaging experiment depicted in **Extended Data Fig 7a** (n = 2). **f** Luminescence-based CETSA experiment depicted as normalised luminescence to each

treatment at 37˚C. K562 BLK-Nluc-3xFLAG cells were treated for 2h with TAK243 followed by 1h DMSO or 10 µM TAK285. T^M: DMSO = 44.60˚C, TAK285 = 44.43˚C; Hill-slopes: DMSO = -20.16, TAK285 = -18.52; R2 value: DMSO = 0.9829, TAK285 = 0.9718; no significant difference, multiple unpaired t-tests (n = 3). Values are shown as mean of the replicates ± SD. **g** ZLyte' *in vitro* inhibition assay data for BLK (m = 2). **h** Kinobead profiling data for the three hits identified for TAK285 in the standard 5-cell line lysate mix. The relative LFQ intensity is depicted on the y-axis and the drug concentration on the x-axis. Following K_Ds were determined: EGFR = 290 nM, ERCC2 = 4.70 µM, MAP2K5 = 2.55 µM. Data depicts the mean ± CI. n = biological replicates, m = technical replicates.

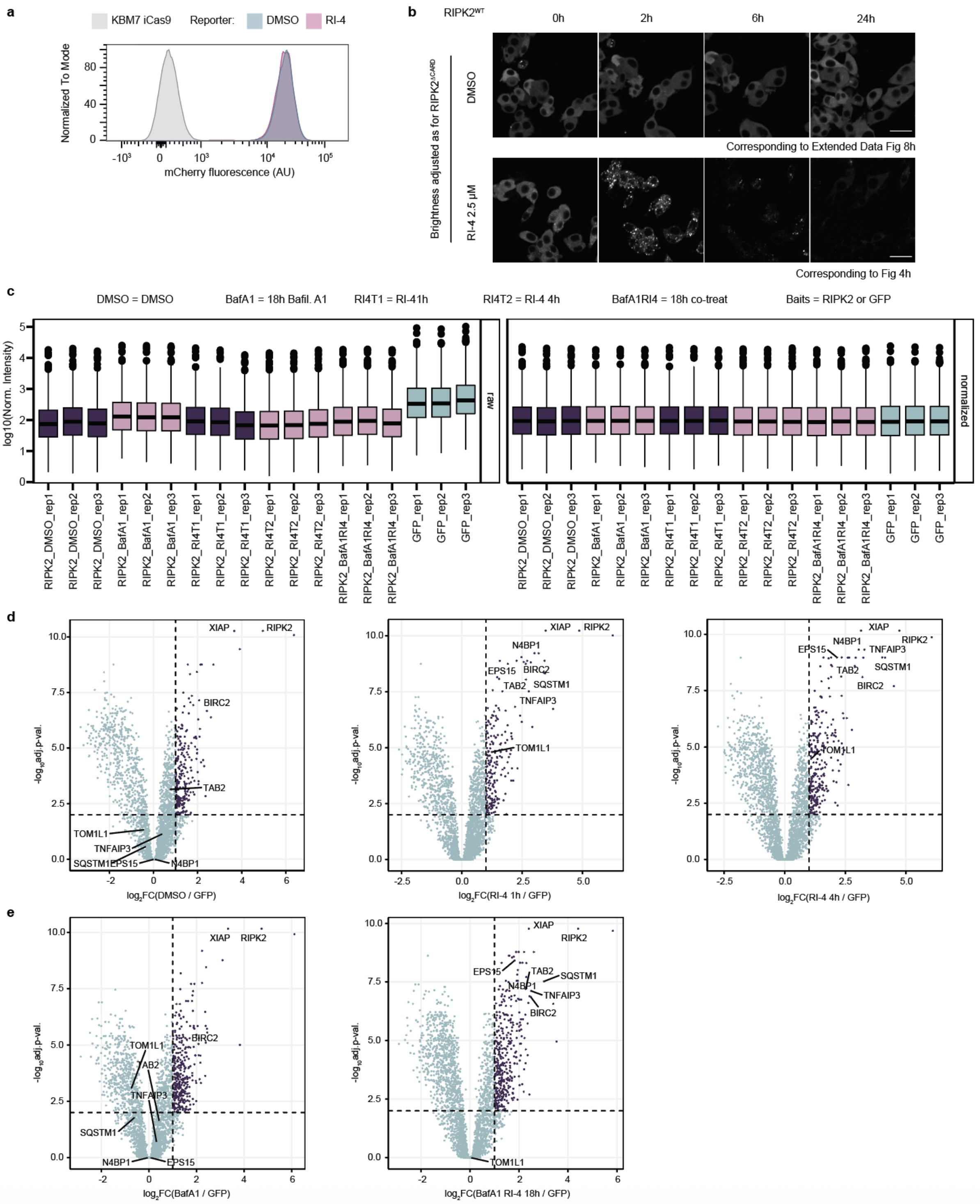

Supplementary Fig S8 Additional data to RIPK2 datasets. **a** Flow histogram matched to **Fig 4a** showing no change of mCherry fluorescence upon RI-4 treatment (18h, 2.5 µM). **b** Data matched to the indicated figure panels with adjusted brightness values equal to the RIPK2$^{\Delta CARD}$ images (n = 3). **c** Distribution of protein intensities ($\log_{10}$) before and after total ion current (TIC) normalisation across RIPK2 and GFP negative controls including baseline (DMSO) and inhibitor treated conditions. Solid line represents the median, box limits show the IQR and its whiskers 1.5 x IQR. Outliers are visualized as black dots (n = 3). **d** Accompanying volcano plots for BioID datasets for single drug treatments (DMSO, 1h 2.5 µM RI-4 or 4h 2.5 µM RI-4) (n = 3). **e** Accompanying volcano plots for BioID datasets for Bafil. A1 (BafA1) co-treated cells (18h of DMSO or 2.5 µM RI-4) (n = 3).

Supplementary References

1. Teo, G. et al. SAINTq: Scoring protein-protein interactions in affinity purification - mass spectrometry experiments with fragment or peptide intensity data. Proteomics 16, 2238–2245 (2016).
