# Supplementary material for: Inhibitors supercharge kinase turnover through native proteolytic circuits
Source: Nature. 2025 Nov 26;649(8098):1032–41. doi: 10.1038/s41586-025-09763-9 (PMC12823440; doi:10.1038/s41586-025-09763-9)
Supplement: Supplementary file 2 — Reporting Summary [file 41586_2025_9763_MOESM2_ESM.pdf]

nature portfolio

Corresponding author(s): Georg E. Winter

Last updated by author(s): Sep 17, 2025

Reporting Summary

Nature Portfolio wishes to improve the reproducibility of the work that we publish. This form provides structure for consistency and transparency in reporting. For further information on Nature Portfolio policies, see our Editorial Policies and the Editorial Policy Checklist.

Statistics

For all statistical analyses, confirm that the following items are present in the figure legend, table legend, main text, or Methods section.

n/a	Confirmed	
☐	☒	The exact sample size (*n*) for each experimental group/condition, given as a discrete number and unit of measurement
☐	☒	A statement on whether measurements were taken from distinct samples or whether the same sample was measured repeatedly
☐	☒	The statistical test(s) used AND whether they are one- or two-sided<br>*Only common tests should be described solely by name; describe more complex techniques in the Methods section.*
☒	☐	A description of all covariates tested
☐	☒	A description of any assumptions or corrections, such as tests of normality and adjustment for multiple comparisons
☐	☒	A full description of the statistical parameters including central tendency (e.g. means) or other basic estimates (e.g. regression coefficient) AND variation (e.g. standard deviation) or associated estimates of uncertainty (e.g. confidence intervals)
☐	☒	For null hypothesis testing, the test statistic (e.g. *F*, *t*, *r*) with confidence intervals, effect sizes, degrees of freedom and *P* value noted<br>*Give P values as exact values whenever suitable.*
☒	☐	For Bayesian analysis, information on the choice of priors and Markov chain Monte Carlo settings
☒	☐	For hierarchical and complex designs, identification of the appropriate level for tests and full reporting of outcomes
☒	☐	Estimates of effect sizes (e.g. Cohen's *d*, Pearson's *r*), indicating how they were calculated

Our web collection on statistics for biologists contains articles on many of the points above.

Software and code

Policy information about availability of computer code

Data collection	NanoGlo Lytic: Multilabel Plate Reader Platform Victor X3 model 2030 (PerkinElmer).<br>Drug screen: EnVision plate reader (Revvity).<br>Flow cytometry: BD LSRFortessa using BD FACSDiva software (v9.0), BD FACSAria Fusion using BD FACSDiva software (v8.0.2) or CytoFLEX SRT Benchtop Cell Sorter using CytExpert SRT-Software (v1.1.0.10007).<br>Western blotting/SDS gels: ChemiDoc Touch imaging system (BioRad) operated on Image Lab (v2.4.0.03).<br>NGS: HiSeq 3000 or NovaSeq 6000, Illumina (https://www.illumina.com/).<br>Mass spectrometry: Orbitrap Fusion Lumos Tribrid mass spectrometer coupled to a Dionex Ultimate 3000 RSLCnano system and operated via Xcalibur (4.3.73.11) and Tune (v3.4.3072.18).<br>Confocal microscopy: PerkinElmer Opera Phenix using Harmony (v4.9 and later versions).<br>MST: Monolith NT.115 (NanoTemper), MO.Affinity Analysis (v2.3)<br>Chemistry: Interchim puriFlash XS 420Plus (column chromatography), Agilent 1260 Infinity II (HPLC), InfinityLab LC/MSD (LR-MS), Bruker Daltonik micrOTOF-QII (HR-MS), Bruker AV 400 HD (NMR)
Data analysis	Flow Cytometry Analysis: Flowjo (v10.6.2)<br>FACS-based CRISPR screens: pipelines for sgRNA quantification and statistical analysis are available on Github (https://github.com/ZuberLab/crispr-processnf/ tree/566f6d46bbcc2a3f49f51bbc96b9820f408ec4a3 and https://github.com/ZuberLab/crisprmageck- nf/tree/c75a90f670698bfa78bfd8be786d6e5d6d4fc455). Packages: fastx-toolkit (v0.0.14), Bowtie2 (v2.4.5), featureCounts (v2.0.1), MAGeCK (v0.5.9).<br>Western blot quantification: Image Lab (v6.1 build 7)<br>Image analysis: cellpose (0.6.5-foss-2020b), cellprofiler (4.1.3-foss-2020b), Fiji (ImageJ, 2.1.1/1.53i)<br>Data compiling, processing and statistical analyses: Microsoft Excel for Microsoft 365 (v16.86), R (v4.3.1), GraphPad Prism (v10.0.3), python (3.7.6), sklearn (v1.0.1), matplotlib (v3.5.3, v3.4.2), seaborn (v0.12.2), numpy (v1.21.5), pandas (v1.0.1), scipy (v1.4.1)

Mass Spectrometry: Proteome Discoverer (v2.4.1.15), MaxQuant (1.5.3.30/v2.4.9.0) , R (version 4.3.1), drc (10.1126/science.ade3925)
DMS: samtools (v1.17, v1.15.1), cutadapt (v4.4), FastqToSam (v3.0.0), Trim Galore (v0.6.6), bwa (v0.7.17), GATK (v4.1.8.1), pheatmap (v.1.0.12), R (v.4.1.0)
Structural predictions and molecular dynamics simulations: AlphaFold3 (V.3.0), CHARMM-GUI Membrane Builder, GROMACS (2023.2), GetContacts (https://getcontacts.github.io/), VMD 1.9.4

For manuscripts utilizing custom algorithms or software that are central to the research but not yet described in published literature, software must be made available to editors and reviewers. We strongly encourage code deposition in a community repository (e.g. GitHub). See the Nature Portfolio guidelines for submitting code & software for further information.

Data

Policy information about availability of data

All manuscripts must include a data availability statement. This statement should provide the following information, where applicable:

- Accession codes, unique identifiers, or web links for publicly available datasets
- A description of any restrictions on data availability
- For clinical datasets or third party data, please ensure that the statement adheres to our policy

Data associated with the drug screening such as hit scores as well as data associated with compounds or kinases have been made available as Supplementary Data 1. Drug screening data have been deposited at https://science.aithyra.at/KinDegData. Additional CRISPR/Cas9 screening data generated in the revision process has been deposited alongside. All processed sequencing and proteomics data has been made available as Supplementary Data 2-7. Additionally, the proteomics data have been deposited to the ProteomeXchange Consortium via the PRIDE partner repository with the dataset identifiers PXD062184 for the in-vivo biotinylation experiments, PXD053130, and PXD059599 for the full proteome profiling and PXD064676 for the TAK285 chemoproteomics. Human protein fasta files were retrieved from UniProtKB (Taxonomic identified 9606, status reviewed, downloaded on the 01.12.2019 or 29.04.2024, https://www.uniprot.org/) and have been deposited alongside the respective MS data. The Kinobeads data have been deposited to the ProteomeXchange Consortium via the MASSIVE partner repository with the data set identifier MSV000095265 alongside with the utilized human protein fasta files (UniProtKB,Taxonomic identified 9606, status reviewed, downloaded on the 22.03.2016).

Research involving human participants, their data, or biological material

Policy information about studies with human participants or human data. See also policy information about sex, gender (identity/presentation), and sexual orientation and race, ethnicity and racism.

Reporting on sex and gender	N/A
Reporting on race, ethnicity, or other socially relevant groupings	N/A
Population characteristics	N/A
Recruitment	N/A
Ethics oversight	N/A

Note that full information on the approval of the study protocol must also be provided in the manuscript.

Field-specific reporting

Please select the one below that is the best fit for your research. If you are not sure, read the appropriate sections before making your selection.

☒ Life sciences ☐ Behavioural & social sciences ☐ Ecological, evolutionary & environmental sciences

For a reference copy of the document with all sections, see nature.com/documents/nr-reporting-summary-flat.pdf

Life sciences study design

All studies must disclose on these points even when the disclosure is negative.

Sample size	All presented data is based on cultured human cell lines. Sample sizes were not predetermined using statistical analyses. Sample sizes were based on prior experience in the field and our previous studies (Mayor-Ruiz et al, Mol Cell, 2019; Mayor-Ruiz et al, Nat Chem Biol, 2020).
Data exclusions	Individual drug screening trajectories were excluded as detailed in the methods.
Replication	Unless stated otherwise in figure legends or method sections, all experiments were done at least twice to ensure reproducibility. The number of independent biological experiments and technical replicates are specified in the respective figure legends.
Randomization	No animal or behavioral studies were conducted. Randomization was thus not necessary. All experiments were conducted in the presence of suitable positive and/or negative controls as indicated.

Blinding | No blinding was performed, as no subjective measurements were done.

Reporting for specific materials, systems and methods

We require information from authors about some types of materials, experimental systems and methods used in many studies. Here, indicate whether each material, system or method listed is relevant to your study. If you are not sure if a list item applies to your research, read the appropriate section before selecting a response.

Materials & experimental systems

n/a	Involved in the study
☐	☒ Antibodies
☐	☒ Eukaryotic cell lines
☒	☐ Palaeontology and archaeology
☒	☐ Animals and other organisms
☒	☐ Clinical data
☒	☐ Dual use research of concern
☒	☐ Plants

Methods

n/a	Involved in the study
☒	☐ ChIP-seq
☐	☒ Flow cytometry
☒	☐ MRI-based neuroimaging

Antibodies

Antibodies used

Immunoblotting: GAPDH (Santa Cruz Biotechnology, sc-365062), GAPDH (Santa Cruz Biotechnology, sc-47724), Vinculin (Szabo Scandic, SACSC-25336), FLAG (Cell Signalling Technology, #2368), LYN (Cell Signalling Technology, #2796), BLK (Cell Signalling Technology, #3262), RIPK2 (Cell Signalling Technology, 4142S), Phospho-Lyn (Tyr507) (Cell Signalling Technology, #2731), FIP200 (Cell Signalling Technology, #12436), CDK9 (Cell Signalling Technology, #2316), TMUB1 (Abcam, EPR14066), cCBL (Cell Signalling Technology, #2747), Phospho-LYN (Tyr397) (Cell Signalling Technology, #70926), HRP-conjugated Anti-Biotin (Cell Signalling, #7075), Peroxidase-conjugated Goat Anti-Rabbit IgG (Jackson ImmunoResearch 111-035-003), Peroxidase-conjugated Goat Anti-Mouse IgG (Jackson ImmunoResearch JAC115035003).

Flow cytometry and FACS: APC anti-mouse CD90.1/Thy-1.1 antibody (1:400, no. 202526, BioLegend) and Human TruStain FcXTM Fc Receptor Blocking Solution (1:400, no. 422302, BioLegend).

Validation

LYN (Fig 2b, degradation assay)
pLYN (Tyr507 and Tyr 397) (Ext Fig 5h, functional assay)
BLK (Ext Fig 6f, degradation assay)
CDK9 (Ext Fig 8e, degradation assay)
FIP200 (Fig 4f, knock-out)
RIPK2 (Fig 4f, degradation assay)
TMUB1 (knock-out, data not shown)
cCBL (Ext Fig 4j, knock-out)
Anti-Biotin-HRP was validated by internal control experiments either without Biotin and/or without doxycycline induction of miniTurbo expression (data not shown).
Validations and multiple hundred references for Vinculin, GAPDH, FLAG and Peroxidase-conjugated AffiniPure Goat Anti-Rabbit IgG or Anti-Mouse can be found on the vendor sites and have been used in multiple, previous in-house studies (e.g. 10.1038/s41589-020-0594-x).
Target specificity for APC anti-mouse CD90.1/Thy-1.1 antibody (no. 202526, BioLegend) was verified by ectopic overexpression (data not shown) and as previously reported (10.1038/s41586-024-07089-6).

Eukaryotic cell lines

Policy information about cell lines and Sex and Gender in Research

Cell line source(s)

KBM7 cells were obtained from T. Brummelkamp lab (Carette et al, Science, 2009), KBM7 iCas9 (originally obtained from Haplogen Bioscience), RKO iCas9-GFP and iCas9-BFP were gifted by J. Zuber (IMP - Research Institute of Molecular Pathology), NALM-6 were obtained from A. Villunger (10.1126/sciadv.ado6607). 293T and K562 were purchased from ATCC, 293T lentiviral packaging cells were obtained from Clontech and Flp-In™ T-REx™ 293 were obtained from Invitrogen. Jurkat cells (Clone E6.1), MCF-7, COLO-205 and MV-4-11 have been previously used in-house (10.1038/s41589-023-01459-3) or obtained from ATCC.

Authentication

All used cell lines were authenticated by vendors and routinely authenticated via cell morphology. Successfull CRISPR-based editing of cell lines was confirmed by cell-based degradation assays and/or immunoblotting. NALM-6 were additionally validated by STR profiling.

Mycoplasma contamination

All used cell lines were routinely tested and confirmed negative for mycoplasma contamination.

Commonly misidentified lines
(See ICLAC register)

No commonly misidentified cell lines were used.

Plants

Seed stocks	N/A
Novel plant genotypes	N/A
Authentication	N/A

Flow Cytometry

Plots

Confirm that:

☒ The axis labels state the marker and fluorochrome used (e.g. CD4-FITC).

☒ The axis scales are clearly visible. Include numbers along axes only for bottom left plot of group (a 'group' is an analysis of identical markers).

☒ All plots are contour plots with outliers or pseudocolor plots.

☒ A numerical value for number of cells or percentage (with statistics) is provided.

Methodology

Sample preparation	The detailed generation and sample preparation can be found in the SI Methods. In brief, stability reporter cell lines were generated by lentiviral transduction of the respective kinase-BFP-P2A-mCherry vectors. Next, cells were either sorted or used directly for experiments. For CRISPR/Cas9 screens and most canonical kinase reporter cell lines, sorted cell pools were additionally sorted as single clones and used after recovery and validation of degradation with the respective hit compounds. For experiments with sorted or unsorted pools of mutated kinase reporters, the respective canonical unsorted or sorted cell pools were used as a reference. sgRNA's were transduced by lentiviral delivery and used after selection with G418. sgRNA harboring cells were confirmed before and after selection by surface antigen staining. For functional degradation experiments, if required gene knock-outs were induced at the timepoints indicated in SI Table S3, and prior to flow cytometry cells were treated with the compounds indicated in the corresponding figure legends. In most instances, cells were directly measured on a BD LSRFortessa. For sorting, cells were stained and fixed as described in the methods and sorted to achieve at least 500 x or 1000 x sgRNA representation per replicate for the genome-wide or UPS focussed sgRNA libraries, respectively. The DMS screen was sorted at >1000 x representation per replicate.<br><br>Flow cytometric data analysis was performed in FlowJo v10.6.2. BFP and mCherry mean fluorescence intensity (MFI) values were normalized by background subtraction of the respective values from reporter-negative KBM7 iCas9 cells. Kinase stability was calculated as the ratio of background subtracted BFP to mCherry MFI, and is displayed normalized to the respective control condition as indicated in the figure legends. For the analysis only reporter positive cells were considered.
Instrument	Data acquisition was performed on a BD LSRFortessa (4 laser, 16 detector configuration; BD Bioscience). Cell sorting was performed on a BD FACSAria Fusion (5 lasers, 16 detectors; BD Bioscience) for CRISPR/Cas9 screens or a CytoFLEX SRT (4 lasers, 15 detectors; Beckman Coulter) for cell line generation.
Software	BD FACSDiva software (v8.0.2 and v9.0), Beckman Coulter CytExpert SRT (v 1.1.0.10007), Flowjo (v10.6.2)
Cell population abundance	For the FACS-based CRISPR/Cas9 screens, cells were sorted into high (5% of cells) or low (5%), and mid (30%) populations. Fractions were re-analyzed after collection for purity. In the case of > 5% of cross- contamination, samples were discarded before further processing. The identical process was applied for the FACS-based DMS screen.
Gating strategy	The forward scatter area vs. side scatter area plot was used to separate cell events from debris and dead cells. Forward scatter height vs. forward scatter area and/or side scatter width vs. side scatter height plots were used to separate single cells from aggregates. For cell populations that had not been sorted prior to the experiments (unsorted pools) or sorted pools with residual reporter negative cells, reporter positive cells were further gated in the Pacific Blue-A (BFP) vs PE-TexasRed-A (mCherry) scatter plots. For the sorting of fixed cells in the CRISPR/Cas9 stability screens, dead cells were excluded based on Zombia-NIR staining (BV786-A) vs FSC-A. Next, triple positive sgRNA (AF 647-A), iCas9-GFP (FITC-A/AF 488-A) and reporter (PE-TexasRed-A) cells were sorted into the respective low, high, and mid populations based on the BFP (BV421-A/Pacific Blue-A) vs mCherry (PE-TexasRed-A) scatter plots. These gates were dynamically adjusted to keep the percentage at 5% for high and low and 30% for MID populations.<br><br>A figure exemplifying the gating strategy for all flow cytometry experiments and FACS-based screens in provided in SI Fig S2.

☒ Tick this box to confirm that a figure exemplifying the gating strategy is provided in the Supplementary Information.
